# Two-dimensional nanomaterials beyond graphene for antibacterial applications: current progress and future perspectives

**DOI:** 10.7150/thno.39701

**Published:** 2020-01-01

**Authors:** Linqiang Mei, Shuang Zhu, Wenyan Yin, Chunying Chen, Guangjun Nie, Zhanjun Gu, Yuliang Zhao

**Affiliations:** 1 CAS Key Laboratory for Biomedical Effects of Nanomaterials and Nanosafety, Institute of High Energy Physics, Chinese Academy of Sciences, Beijing 100049, China.; 2 CAS Center for Excellence in Nanoscience, National Center for Nanoscience and Technology of China, Chinese Academy of Sciences, Beijing 100190, China.; 3 College of Materials Science and Optoelectronic Technology, University of Chinese Academy of Sciences, Beijing 100049, China.

**Keywords:** bacterial resistance, antibacterials, 2D NBG, antibacterial mechanisms, physicochemical properties

## Abstract

The marked augment of drug-resistance to traditional antibiotics underlines the crying need for novel replaceable antibacterials. Research advances have revealed the considerable sterilization potential of two-dimension graphene-based nanomaterials. Subsequently, two-dimensional nanomaterials beyond graphene (2D NBG) as novel antibacterials have also demonstrated their power for disinfection due to their unique physicochemical properties and good biocompatibility. Therefore, the exploration of antibacterial mechanisms of 2D NBG is vital to manipulate antibacterials for future applications. Herein, we summarize the recent research progress of 2D NBG-based antibacterial agents, starting with a detailed introduction of the relevant antibacterial mechanisms, including direct contact destruction, oxidative stress, photo-induced antibacterial, control drug/metallic ions releasing, and the multi-mode synergistic antibacterial. Then, the effect of the physicochemical properties of 2D NBG on their antibacterial activities is also discussed. Additionally, a summary of the different kinds of 2D NBG is given, such as transition-metal dichalcogenides/oxides, metal-based compounds, nitride-based nanomaterials, black phosphorus, transition metal carbides, and nitrides. Finally, we rationally analyze the current challenges and new perspectives for future study of more effective antibacterial agents. This review not only can help researchers grasp the current status of 2D NBG antibacterials, but also may catalyze breakthroughs in this fast-growing field.

## 1. Introduction

The diseases caused by bacterial infections continue to be a major growing global health issue. According to statistics, pathogenic microorganisms including bacteria, fungi, viruses, protozoa, prions, and rickettsia caused millions of deaths all over the world annually 1-3. Subsequently, the advent of antibiotics has effectively inhibited bacterial infections and saved countless lives, which are attributed to their natural properties and abilities to selectively kill bacteria by disrupting cellular processes including protein synthesis, deoxyribonucleic acid (DNA) replication/repair, and cell-wall/membrane formation 4-7. However, the overuse or misuse of traditional antibiotics has resulted in the emergence and rapid increase of multi-drug resistance “superbugs” day after day, consequently leading to extremely severe side effects 8-10. It is estimated that if antibiotics resistance continues to emerge, antibiotic-resistant infections will cause over ten million deaths annually and considerable global economy loss 11-13. In addition, the formation of biofilm is another major obstacle that affects the bactericidal efficiency of traditional antibiotics 14-17. Therefore, it is an urgent demand to search for low toxic antibiotics or develop new classes of alternative antibacterial to alleviate or address the looming crisis of bacterial resistance 18-21.

Recently, sparked by the rapid development of nanoscience and technology, substantial efforts have been aimed to develop versatile nanomaterials as novel antibacterial against various bacterial pathogens based on their extraordinary physicochemical characteristics 22-25. Compared to traditional antibiotics, nanomaterials are less likely to trigger bacterial resistance owing to their good membrane permeability, benign biocompatibility and potential for multiple antibacterial actions 26-28. In addition, 2D nanomaterials with large surface area and easy surface functionalization enable intimate interactions with bacteria membranes, which help in enhancing the antibacterial effect. Moreover, the 2D nanomaterials based antibacterial agents can be used at a low dose than traditional antibiotics, hence overcoming the problem of resistance and diminishing other undesirable side effects to some extent 29-31. Significant progress has been achieved on the development of nanotechnology-based antibacterial nanoagents (graphene 32, noble metals 33, organic polymers 34, *etc.*) for combating multidrug resistance in bacteria through physical contact damage 35, oxidative stress 36, photo-induced antibacterial (such as photothermal therapy (PTT) 37, photocatalytic therapy (PTC) 38, and photodynamic therapy (PDT) 39), controlled drug/metallic ions releasing 40, and multi-mode synergistic antibacterial 41. Among various antibacterial nanoagents, 2D graphene and its derivatives have exhibited attractive applications in biomedicine (tumor diagnosis and therapy, neurological disorders treatment, and antibacterial) over the past decade 42-47, because of their intriguing biochemical properties, such as easy preparation and functionalization, high specific surface area, and good biocompatibility 48, 49. Moreover, the investigations of graphene-based nanomaterials also facilitated the exploration of new two-dimensional nanomaterials beyond graphene (2D NBG) 50. Fortunately, in recent years, 2D NBG has displayed attractive applications in the fields of catalysis, optical/electronic devices, and biomedicine, which could be attributed to favorable graphene-like physicochemical characteristics, such as large specific surface-area, ultrathin 2D nature, striking light-to-heat capability, extraordinary photocatalytic features, facile surface modification, and relatively benign biocompatibility 51-53. Particularly, recent advances reveal that 2D NBG has a robust antibacterial effect 54. Moreover, these graphene-like nanomaterials-oriented antibacterials presented different types of antibacterial mechanisms 55. To date, the family of 2D NBG nanomaterials have been enriched by many members, which include 2D layered transition metal dichalcogenides (TMDCs), transition metal oxides (TMDOs), graphitic carbon nitride (*g*-C_3_N_4_), black phosphorus (BP), layered double hydroxides (LDHs), transition metal carbides and nitrides (MXenes), and so on 52. Currently, broad researches have been pursued on antibacterial applications of 2D NBG, such as direct interaction mechanism between 2D NBG and bacteria 56. However, a comprehensive review with antibacterial progress, size/shape/phase structure and surface modification-oriented antibacterial advantages, future perspectives and challenges aiming specifically at 2D NBG for antibacterial is still rare. Therefore, it is obligatory to present a systematically summary of the recent research progress of 2D NBG in antibacterial field.

**Figure 1:**
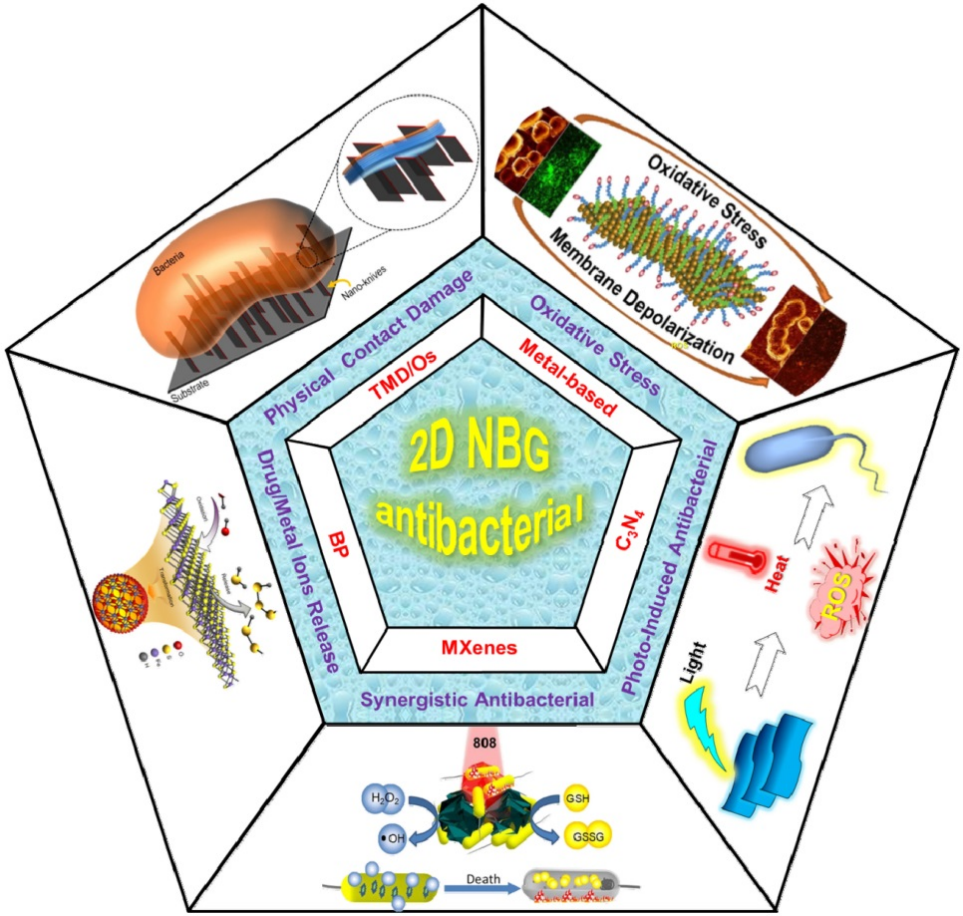
The existing antibacterial mechanisms and kinds of 2D NBG. Physical contact damage: Reproduced with permission from 58, copyright 2018 American Chemical Society. Oxidative stress: Reproduced with permission from 83, copyright 2016 American Chemical Society. Photo-induced generate heat or ROS for antibacterial. Controlled drug/metallic ions release: Reproduced with permission from 134, copyright 2018 Nature Publishing Group. Multi-mode Synergistic antibacterial: Reproduced with permission from 143, copyright 2016 American Chemical Society.

Herein, we provide a critical overview on the recent advances of 2D NBG-based antibacterial agents, specifically focusing on their dominant antibacterial mechanisms (schematic illustration in **Figure 1**). We first outline the underlying antibacterial mechanisms of 2D NBG and their derivatives as antibacterials based on the existing research studies. Then, a brief discussion of the relationship between the physicochemical characteristics of 2D NBG and antibacterial efficiency is presented. Furthermore, we enumerate some representative 2D NBG and their relevant antibacterial action. Finally, we provide our perspectives about the major obstacles in clinical translation of 2D NBG. We hope that this review can help researchers to understand the properties and antibacterial mechanisms of 2D NBG for antibacterial and draw public attention to develop novel efficient antibacterial strategies using the 2D NBG in the future.

## 2. Antibacterial Mechanisms and Physicochemical Characteristics Related Influencing Factors of 2D NBG

To the best of our knowledge, graphene-based nanomaterials with outstanding antibacterial activities based on their extraordinary characteristics, and predominant antibacterial mechanisms have been systematically reviewed by Luo's group in 2016 57.

However, a thorough understanding of the underlying antibacterial mechanisms of 2D NBG remains in its infancy. Therefore, it is essential to summarize the existing antibacterial mechanisms of 2D NBG to manipulate antibacterial nanomaterials for future biomedical applications. According to recent achievements, we summarize that current antibacterial mechanisms of 2D NBG mainly include physical contact destruction, oxidative stress, photo-induced antibacterial, controlled drug/metallic ions release, and multi-mode synergistic antibacterial effects 54, 56. In the following section, we will comprehensively clarify each antibacterial mechanism of 2D NBG and offer some hints to improve antibacterial effects.

### 2.1 Physical Contact Destruction

**Figure 2:**
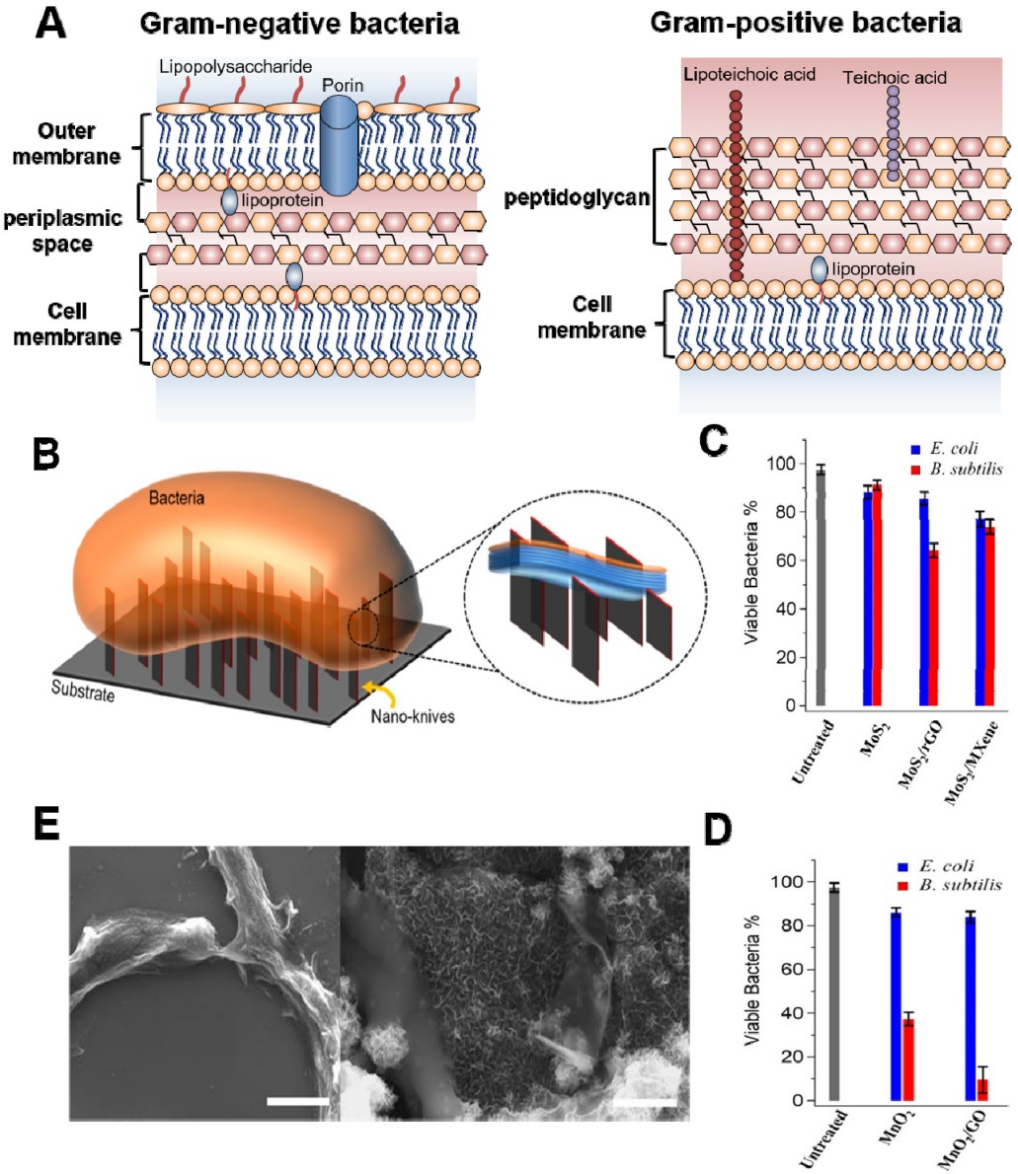
(**A**) General membrane ultrastructure of Gram- and Gram+ bacterial species. (**B**) Schematic representation of direct physical interaction of the bacterial surface with sharp edges of vertically aligned nanosheets onto a substrate. Antibacterial activities of (**C**) MoS_2_ and (**D**) MnO_2_ nanosheets against *E. coli* and *B. subtilis*. (**E**) SEM images of the *B. subtilis* bacteria treated with MnO_2_ and MnO_2_/GO nanomaterials for 3 h in dark. Reproduced with permission from 58, copyright 2018 American Chemical Society.

Several accumulated studies have demonstrated that the cell wall or membrane is an indispensable component in all bacteria, which is accountable for maintaining cell morphology, regulating osmotic pressure, and preventing infection 54. It has been verified that Gram- and Gram+ bacteria have different cell membrane structure (**Figure 2A**) 58-60. The former possesses twain distinct lipoprotein membranes: a lipopolysaccharide coated inner cell membrane and outer membrane, which is separated by a thin layer of peptidoglycan in the periplasmic space 59. Conversely, Gram+ bacteria are composed of single lipid bilayer cell membrane and multilayer thick peptidoglycan mesh outside of the cell membrane 60. It has been confirmed that sharp edges of nanosheets (also regarded as “nanoknives”) were probably compromising bacterial membrane integrity upon physical contact, which could lead to the leakage of intracellular components, such as ribonucleic acid (RNA), DNA, phospholipids, and proteins,* etc.* 61. This mechanism was initially proposed for graphene-based nanomaterials 35, 62. In recent years, various 2D NBG such as MoS_2_, WS_2_, MoO_3_, RuO_2_, MnO_2_, and Bi_2_Se_3_ have been used for physical contact disinfection because their sharp edges could damage cell wall or outer membrane 52. For instance, Alimohammadi *et al.* reported that MnO_2_ and MoS_2_ nanosheets with blade-like shape show remarkable antibacterial effects towards *B. subtilis* and *E. coli* (**Figure 2B**) 58. Intriguingly, they studied the antibacterial activities of randomly oriented and vertically aligned MoS_2_/MnO_2_ nanosheets on graphene oxide or Ti_3_C_2_ MXene nanosheets, and observed that vertically aligned MnO_2_ nanosheets exhibit higher antibacterial activity than randomly oriented nanosheets, which is ascribed to the sharp edges of vertically aligned MnO_2_ nanosheets drastic damaging the integrity of bacterial cell membrane (**Figure 2C, D**). Meanwhile, scanning electron microscope (SEM) images of the *B. subtilis* also verified that the sharp edge of MnO_2_ nanosheets can distinctly destroy bacterial morphology and ultimately kill bacteria, which also proved that 2D antibacterials probably mainly damage the peptidoglycan mesh of bacteria (**Figure 2E**). Similarly, Krishnamoorthy *et al.* revealed that the plate-like morphology of the as-prepared MoO_3_ functioned as “nanoknives” could kill bacteria through physical puncture of the outer bacterial wall 63, 64. Furthermore, Liu *et al.* found that WS_2_ nanosheets cling more bacterial and show a robust antibacterial activity 65. From the SEM and transmission electron microscope (TEM) images, they proposed that WS_2_ nanosheets with sharp edges could apparently damage the structural integrity of the bacterial membrane, which is induced by the direct contact of bacteria and WS_2_ nanosheets. Besides, Jiang *et al.* developed a novel Bi_2_Se_3_ nanodiscs through solvent thermal reaction for selectively treating Gram+ bacterial infections 66. They found that Bi_2_Se_3_ nanodiscs mainly damage bacterial wall/membrane by binding with lipoteichoic acid of Gram+ bacteria. It is worth noting that theoretical simulation results revealed that the physical contact destruction action of graphene nanosheets mainly depends on their sizes 67-70. However, it is not clear whether the size or other physicochemical properties of 2D NBG will affect their antibacterial activity. Therefore, physical contact destruction as one of the most common antibacterial mechanisms should be further studied. And the combination of theoretical simulation with experiment to deeply explore the effect of nanomaterial physicochemical properties on antibacterial activity is also recommended.

### 2.2 Oxidative Stress Antibacterial

Oxidative stress can impede bacterial metabolism and continuously damage essential cellular functions, causing bacteria inactivation, and eventually killing bacteria 71, 72. Therefore, the generation of oxidative stress is one of the widely accepted antibacterial methods. In general, 2D nanomaterials-mediated oxidative stress mainly includes reactive oxygen species (ROS)-dependent and ROS-independent oxidative stress.

#### 2.2.1 ROS-Dependent Oxidative Stress

**Figure 3:**
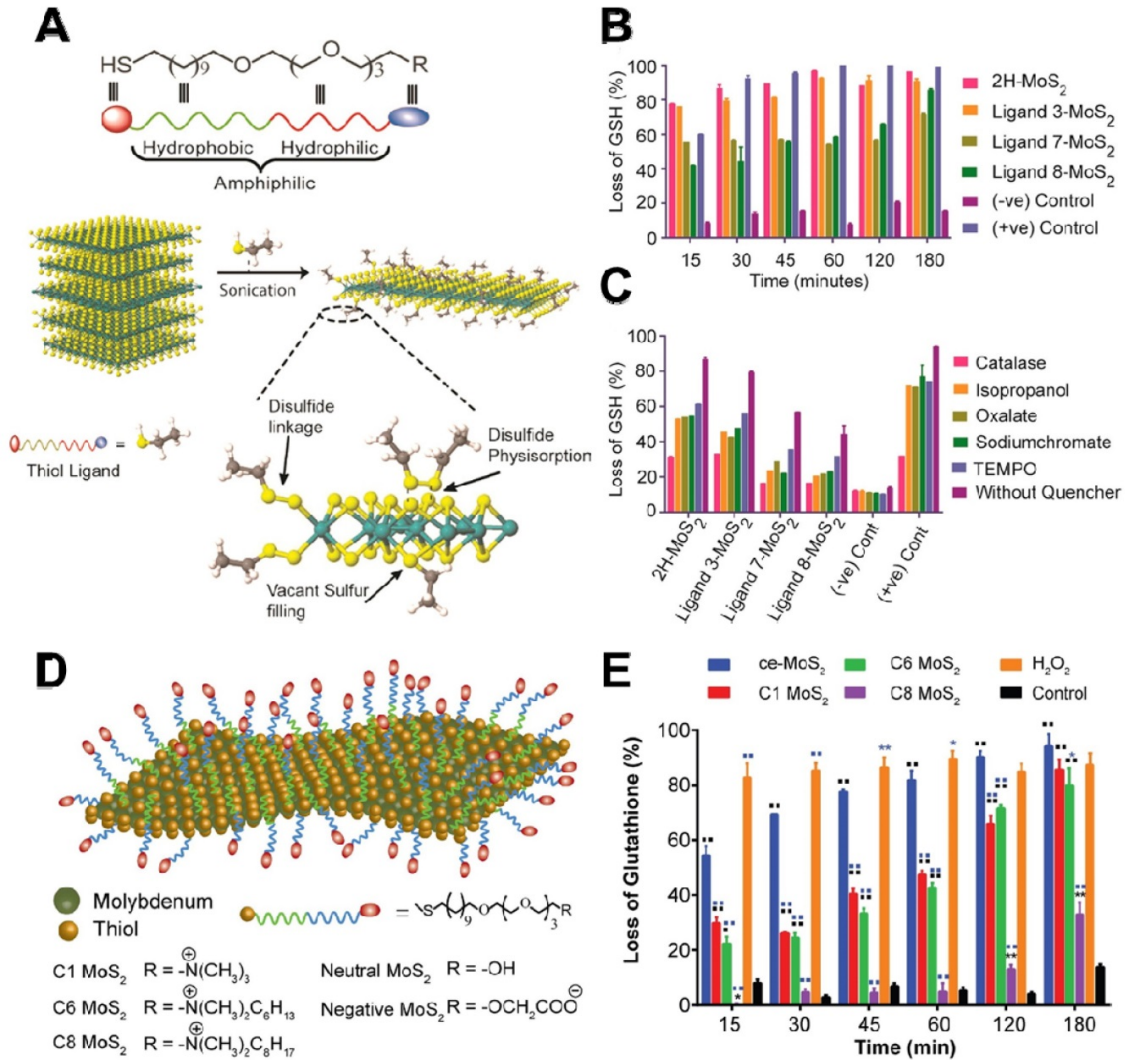
(**A**) Scheme for the exfoliation of 2H-MoS_2_ with amphophilic ligand. (**B**) Abiotic oxidative stress estimation by Ellman's assay with 0.4 mM glutathione. (**C**) Estimation of the type of ROS species in the presence of different ROS scavengers. Reproduced with permission from 74, copyright 2018 American Chemical Society. (**D**) Schematic representation of functionalized ce-MoS_2_ with thiol ligands of varied charge and hydrophobicity. (**E**) Abiotic glutathione oxidation assay for quantification of oxidative stress generated. Reproduced with permission from 83, copyright 2016 American Chemical Society.

ROS-dependent oxidative stress is triggered by excessive accumulation of intracellular ROS, such as hydrogen peroxide (H_2_O_2_), hydroxyl radicals (•OH), superoxide anions (^•^O_2_^-^), and singlet molecular oxygen (^1^O_2_) under external/internal stimulus factors. Previous studies have demonstrated that graphene and its ramifications can generate ROS 8. In general, an excessive amount of ROS not only causes direct damage to the bacterial structure by breaking DNA single strand but also leads to hyperoxidation of intracellular components. Recently, 2D NBG has been found to exert antibacterial performances through the production of ROS under light-dependent or light-independent stimulus factors. Several cumulative studies have documented that 2D MoS_2_ nanosheets with supramolecular selfassembly property could selectively produce ROS in a target cell rather than control cells, which is beneficial for their further biomedical application 73. To verify the ROS-dependent oxidative stress, Karunakaran *et al.* used different thiol surfactant molecules to exfoliate and functionalize 2H-MoS_2_ (H stands for hexagonal) nanosheets for enhancing the antibacterial effect (**Figure 3A**) 74. By monitoring ROS levels using Ellman's assay, they found that 2H-MoS_2_ nanosheets with suitable conduction band could incur more loss of glutathione (GSH) when compared to 1T-MoS_2_ (T is trigonal) nanosheets (**Figure 3B**). Subsequently, they used different scavengers to ascertain the ROS type and found that H_2_O_2_ is the dominant ROS (**Figure 3C**). Moreover, they used fluorescent probe 2',7'-dichlorofluorescin diacetate (DCFDA) to estimate the amount of intracellular ROS, and the results revealed that positively charged 2H-MoS_2_ nanosheets can effectively attach to the bacterial surface and generate more ROS. In contrast, the negatively charged 2H-MoS_2_ nanosheets with non-interactive property produced less ROS in bacteria. Therefore, due to the different semiconducting property of 2H and 1T phase, when compared to 1T-MoS_2_, the ROS-dependent oxidative stress is more obvious in 2H-MoS_2_ nanosheets. Furthermore, the higher antibacterial capacity of exfoliated 1T-phase TMDCs (including MoS_2_, WS_2_, MoSe_2_) nanosheets is also attributed to the production of ROS 75, 76. In addition, Xiong *et al.* demonstrated that BP nanosheets can also strikingly kill *E. coli* and *B. subtilis* through ROS-dependent oxidative stress and membrane damage mechanism 77. These studies not only partially verified the generation of ROS in bacteria, but also proved that ROS-dependent oxidative stress is an important antibacterial process.

#### 2.2.2 ROS-Independent Oxidative Stress

Although the above-mentioned reviews underline the crucial role of ROS-dependent oxidative stress, several studies have revealed that ROS-independent oxidative stress also possesses a favorable bactericidal effect. In detail, ROS-independent oxidative stress mainly damages or oxidizes cellular structure and components (such as lipids, proteins, and DNA) by nanomaterials-induced direct oxidation rather than ROS production 78, 79. GSH, a ubiquitous antioxidant *in vivo*, not only can serve as an intracellular redox state indicator but also can indicate the oxidative stress antibacterial effect owing to its easy oxidation into glutathione disulfide (GSSG) 80. In 2008, Alvarez's group firstly proposed this antibacterial mechanism and they observed that the antibacterial ability of fullerene (nC_60_) does not rely on light and oxygen, which are the two conditions essential for ROS generation 81. Similarly, Yang *et al.* used a chemically exfoliated (ce) method to synthesize ce-MoS_2_ nanosheets and studied their antibacterial activity 82. They found that ce-MoS_2_ nanosheets could dramatically reduce the viability of *E. coli* in a short time, and show concentration- and time-dependent GSH oxidation capacity. Therefore, the antibacterial activity of ce-MoS_2_ nanosheets is attributed to ROS-independent oxidative stress. In addition, Mrinmoy's group revealed that the surface charge and hydrophobicity of ce-MoS_2_ nanosheets can be altered by modifying with different thiol ligands (**Figure 3D**), and such functionalized ce-MoS_2_ nanosheets can be endowed with remarkable antibacterial effect by ROS-independent oxidative stress and depolarization of the bacterial membrane 83. Intriguingly, ce-MoS_2_ nanosheets showed higher GSH oxidizability than functionalized MoS_2_ nanosheets (**Figure 3E**), which is responsible for the ROS-independent oxidative stress generated by ce-MoS_2_ nanosheets. What's more, they also found that the surface functionalization can affect antibacterial pathway, and non-functionalized MoS_2_ nanosheets against bacteria mainly through ROS-induced oxidative stress, while functionalized MoS_2_ nanosheets kill bacterial through both ROS-independent oxidative stress and bacterial membrane depolarization. Although vast research has substantiated the striking germicidal ability of graphene oxide (GO) and MoS_2_ nanosheets, the relevant study of 2D nanosheet-coated films for biomedical devices is still rare. Based on this, Kim *et al.* prepared a GO-MoS_2_ nanocomposite film with outstanding antibacterial effects 84. They found that GO-MoS_2_ nanofilms have higher ROS-independent oxidation capacity, and its antibacterial effect is mostly achieved through ROS-independent oxidative stress. Furthermore, Navale *et al.* also reported that the reduced graphene oxide-tungsten disulphide (rGO-WS_2_) nanosheets had a significant bacterial inhibitory effect compared to pure WS_2_ or rGO. They observed that rGO-WS_2_ composites have the highest oxidation capacity towards GSH, and proposed that the GSH membrane mechanism stress may originate from the direct contact with nanosheets, and ROS-independent oxidation stress was the major antibacterial mechanism 85.

### 2.3 Photo-Induced Antibacterial Effects

It is well-known that light-induced antibacterial mainly utilized photoconversion of excited photoactive fluorophores in photosensitizer, photothermal agents and other semiconductor nanomaterials into heat or ROS for PTT, PCT or PDT 37, 38, 86, 87. Therefore, in recent years, these photo-induced antibacterial methods have been considered as a promising antibacterial mechanism due to their peculiar merits such as noninvasiveness, targeted selective treatment, and minimized side effects.

#### 2.3.1 Photothermal Antibacterial

**Figure 4:**
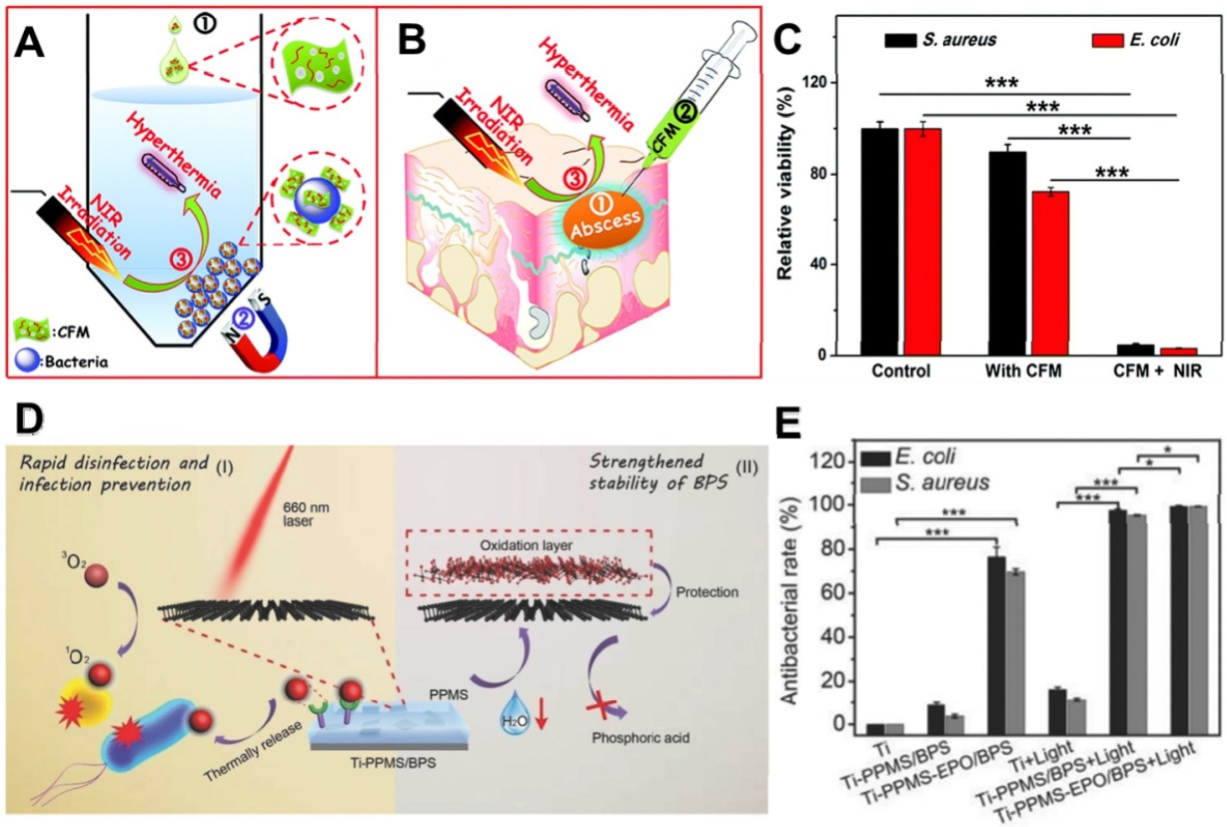
(**A**) *In vitro* conjugating and photothermal killing of bacteria, (**B**) and *in vivo* photothermal treatment of a focal infection using chitosan functionalized magnetic MoS_2_. (**C**) Antibacterial activity of the chitosan functionalized magnetic MoS_2_ nanocomposites with or without NIR laser irradiation for *S. aureus* and *E. coli*. Reproduced with permission from 95, copyright 2016 Royal Society of Chemistry. (**D**) Schematic that shows the PPMS/BPS inactivating bacteria through generating ^1^O_2_ in the presence and absence of light, and the stability of BPS can be improved by PPMS. (**E**) Antibacterial performance *in vitro*. Reproduced with permission from 122, copyright 2018 WILEY-VCH Verlag GmbH & Co. KGaA, Weinheim.

Photothermal antibacterial is an important antibacterial approach, which refers to the efficient generation of heat by nanomaterials under the irradiation of light at a proper power density to kill bacteria 37. Near-infrared (NIR) photothermal nanoagents are preferred for antibacterial due to their deep biological tissue penetration capability and minimal damage to healthy tissues. In recent years, researchers have demonstrated that various 2D NBG display remarkable photothermal antibacterial ability owing to their strong photothermal conversion efficiencies, such as MoS_2_ 88, metal-based nanomaterials 89, Sb_2_Se_3_ 90, LDH-based compounds 91, and BP nanosheets 92. Among them, MoS_2_ nanosheets have attracted considerable attention as typical photothermal antibacterial agents due to their high NIR photothermal conversion efficiency 93, 94. Based on it, Zhang *et al.* prepared versatile chitosan functionalized magnetic MoS_2_ as bacterial cross-linking and photothermal agents for *in vitro* photothermal sterilization (**Figure 4A**), and *in vivo* focal infection treatment (**Figure 4B**) 95. The nano-agent could cause rapid aggregation and bacteria arrest in both *S. aureus* and *E. coli*, thereby enhancing the photothermal antibacterial effect. Under NIR irradiation, the temperature of the nano-agent solution (100 μg mL^-1^) could reach around 45 ^o^C within 10 min, and the bacteria survival rate decreased over 90% (**Figure 4C**). Besides, this nano-agent nanoplatform could also effectively convert NIR light into localized heat for treating *in vivo* focal infection. More recently, Ma *et al.* developed a core-shell gold nanorod@layered double hydroxide (GNR@LDH) nanomaterial for enlarging PTT antibacterial and tumor therapy 91. They found that the thermal energy conversion of GNR@LDH can be significantly enhanced after 808 nm laser irradiation, which is mainly due to the production of electron deficiency on gold surface. As shown above, the burgeoning photothermal nanomaterials with strong NIR light absorbance/convert capacity revealed that the generated heat can not only kill drug-resistant bacteria but also inhibit the formation of biofilm. Therefore, 2D NBG based PTT is deemed as a safe and efficient strategy to combat bacterial infections.

#### 2.3.2 Photocatalytic/Photodynamic Antibacterial

It is well-known that the photo-induced production of ROS could cause distinct oxidative stress to bacteria. Generally, the photo-mediated ROS generation mainly includes two ways: photocatalytic and photodynamic process. Photocatalytic antibacterial refers to light-induced bacteria inactivation, which depends on the efficient generation of highly oxidative ROS by the separation of holes (h^+^) and electrons (e^-^) in the valence band (VB) and conduction band (CB) under light irradiation 96. For many 2D NBG, they can generate photo-induced ROS to attack bacteria under ultra violet (UV) or visible light illumination. Normally, the photocatalytic disinfection process mainly includes three steps: (i) 2D NBG with large surface area can bind to bacterial surface, which provide more active sites for catalytic reactions; (ii) the formation of a photo-induced charge-separated state; (iii) the interactions of activated charge-separated centers and neighboring oxygen and water molecules can produce ROS 97. Bismuth oxybromide (BiOBr) nanosheets, as a major 2D layered semiconductor, have attracted tremendous attention owing to its outstanding physicochemical prosperities and excellent visible light photocatalytic activity 98. To further efficiently harvest solar energy, Wong's group fabricated bismuth oxybromide (BiOBr) nanosheets and doped with boron (B), where they further found that B-BiOBr nanosheets could effectively enhance photocatalytic bacteria inactivation through photogenerated h^+^ mediated mechanism 99. Firstly, light motivated BiOBr to generate e^-^ in its CB, and leave h^+^ in its VB. Then, B as a good e^-^ acceptor could promote an extra e^-^ from VB to CB and cause abundant h^+^ gathering in VB, which could enhance the e^-^/h^+^ pair separation efficiency of BiOBr. Finally, the amount of h^+^ with higher oxidative ability could directly oxidize bacteria, leading to photocatalytic bacteria inactivation. They also found that B-BiOBr nanosheets show apparent photocatalytic disinfection activity towards *E. coli*, and the increased content of B dopant could dramatically enhance the antibacterial properties. Moreover, Wu *et al.* demonstrated that self-doped Bi^5+^ or Bi_2_O_4_ decorated BiOBr nanosheets display a much higher photocatalytic inactivation activity than pure BiOBr nanosheets 100, 101. Recently, g-C_3_N_4_ has been a rising star in the field of photocatalyst disinfection as it could harvest substantial visible light for achieving highly efficient photocatalytic performance 102-104. For example, Huang *et al.* for the first time revealed that g-C_3_N_4_ nanosheets exhibited excellent photocatalytic bactericidal effects toward *E. coli* under visible light irradiation, without any bacterial regrowth 105. Several cumulative studies have documented that exfoliating the bulk g-C_3_N_4_ into g-C_3_N_4_ nanosheets could improve the photocatalytic performance. To this end, Zhao *et al.* fabricated single layer g-C_3_N_4_ nanosheets by thermal etching and ultrasonic exfoliation. They found that a total of 2 × 10^7^ CFU mL^-1^ of *E. coli* could be destroyed completely within 4 h after incubated with single layer g-C_3_N_4_ nanosheets under visible light irradiation, which is much higher than bulk g-C_3_N_4_ nanosheets 106. Besides, some other 2D NBG, such as MoS_2_, TiO_2_, Bi_2_WO_6_, ZnO, as well as metal-free nanomaterials, also exhibited outstanding photocatalytic antibacterial activity 97, 104, 105, 107-109. To further improve the photocatalytic antibacterial activity, the integration of various photocatalysts or co-catalysts, such as Ag-ZnO/g-C_3_N_4_ 110, g-C_3_N_4_ NS/RGO/CA 111, V_2_O_5_/BiVO_4_ 112, graphene/g-C_3_N_4_ 113, TiO_2_/g-C_3_N_4_ 114, TiO_2_-Bi_2_WO_6_ 108, and Bi_2_MoO_6_/g-C_3_N_4_ 115, has been widely investigated as an effective modification strategy. Although photocatalytic disinfection represents one of the most promising disinfection mechanisms, important issues such as catalyst immobilization, recycling methods, photocatalytic reactor design as well as optimization of disinfection parameters of 2D NBG need to be addressed for future practical applications.

In addition to above photocatalytic-induced bacteria elimination, PDT as another light-induced ROS generating modality for minimally invasive antibacterial has also been reported 86. It mainly employs appropriate excitation light and oxygen molecules as exogenous stimuli to selectively activate photosensitizers at the targeted region, producing various cytotoxic ROS like ^1^O_2_ and •OH to effectively damage the bacterial membrane, nucleic acids, proteins, and even DNA. Additionally, PDT can also eliminate biofilms by degrading their excretive products 116. Notably, PDT has several advantages over other conventional antibacterial strategies owing to its noninvasive property, massive loading capability, and fast healing process. PDT can be carried out through two paths: type I and type II. Under light irradiation, photosensitizers migrate from ground state to single excited state, and then reach to triple excited state 117, 118. For type I, the triple excited-state photosensitizers directly react with the biological substrates to generate •OH or ^•^O_2_^-^ through electron transfer, and this process not only break the structural integrity of the bacterial membranes but also enhance ionic permeability of bacteria cell membrane 119. For type II, triple excited-state photosensitizers could directly transfer energy with the surrounding ^3^O_2_ to produce cytotoxic ^1^O_2_, and the ^1^O_2_ can effectively kill bacteria by causing oxidative damages to unsaturated lipids, enzymes, DNA, peptides, and other cellular components 120. Currently, various 2D nanomaterials such as MoS_2_ nanosheets, BP nanosheets, fullerenes, graphene, ZnO, and TiO_2_, show inherent photosensitivity, and they have been widely explored for PDT through generating ROS upon light irradiation 121. Recently, Tan *et al.* designed a novel PDT antibacterial system based on the integration of BP nanosheets and poly (4-pyridonemethylstyrene) (PPMS), achieving the storage and release of ^1^O_2_ for rapid disinfection and infection prevention (**Figure 4D**) 122. Intriguingly, BP nanosheets were used as a photosensitizer to generate ^1^O_2_ under light irradiation. Simultaneously, the PPMS were composited with the BP nanosheets to store singlet ^1^O_2_, and the ^1^O_2_ was released by thermal decomposition. They found that this system showed an excellent PDT antibacterial effect with or without light irradiation (**Figure 4E**). Therefore, 2D-based nano-photosensitizers as light-triggered bactericides show great potential for PDT antibacterial.

### 2.4 Controlled Drug/Metallic Ions Release for Antibacterial

**Figure 5:**
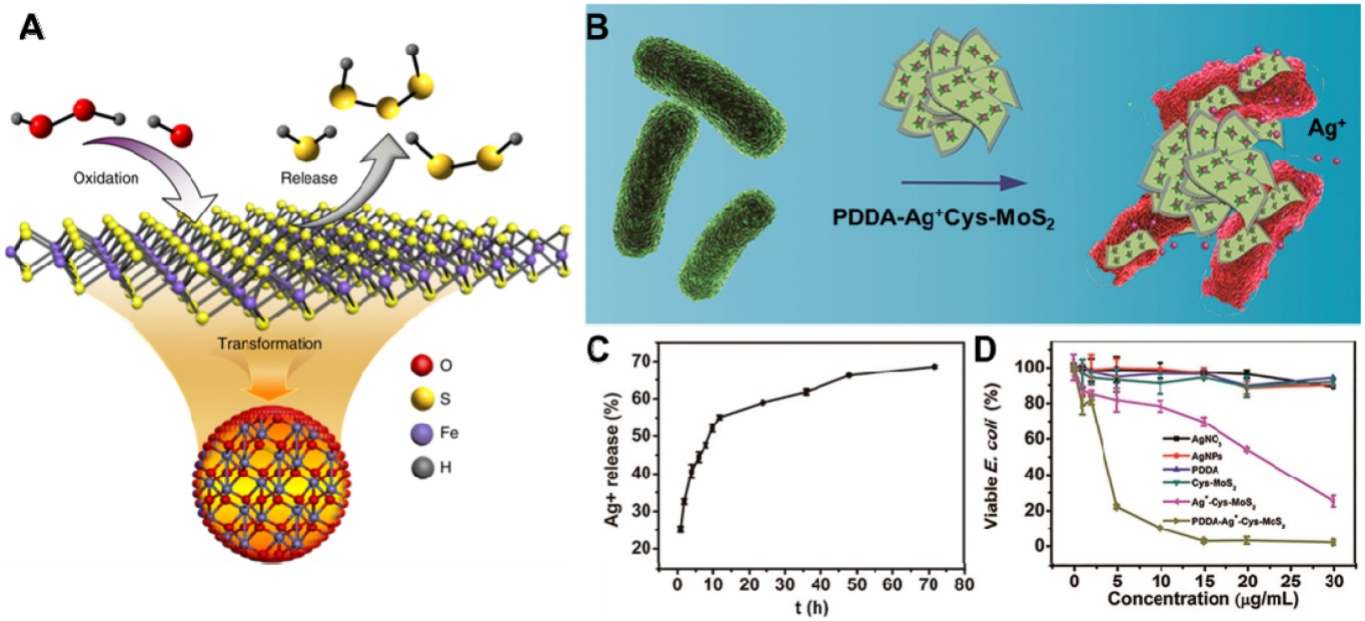
(**A**) Scheme of polysulfane release from nFeS. Reproduced with permission from 134, copyright 2018 Nature Publishing Group. (**B**) The PDDA-Ag^+^-Cys-MoS_2_ depot for antibacterial applications. (**C**) Cumulative silver ion release profiles from PDDA-Ag^+^-Cys-MoS_2_ samples. (**D**) Viability analyses of *E. coli*. Reproduced with permission from 136, copyright 2017 American Chemical Society.

The ability of controllable release drug/metallic ions is widely accepted as an important antibacterial process of 2D NBG 123. Due to their large surface area, 2D NBG can act as nanocarriers with controlled release of antibacterial agents under particular stimuli 124. Currently, the release of antibacterial agents mainly include either converting unstable organosulfur compounds into stable inorganic sulfur compounds 125 or loading traditional antibiotics (cefazolin 126, tetracycline hydrochloride 127, and curcumin *etc.* 128), noble metallic ions (such as Ag ions 129-131) and molecular donor (NO 132, H_2_S donor 133) into 2D NBG surface. Ultimately, released drugs or metallic ions on the surface of 2D NBG can inactivate the bacteria through interfering essential cellular components such as cell membrane integrity, respiration, and adenosine triphosphate (ATP) production. Recently, Gao's group proposed a novel nano-conversion strategy for enhancing antibacterial activity through converting natural organosulfur compounds into nano-iron sulfides (FeS), where the enhanced antibacterial effect benefited from cysteine-nFeS (Cys-nFeS) with enzyme-like activity could effectively increase the release of bactericidal polysulfanes (**Figure 5A**) 134. The antibacterial results also showed that Cys-nFeS not only display a broad antibacterial activity against *Pseudomonas aeruginosa* (*P. aeruginosa*), *E. coli*, and *S. aureus*, but also could effectively inhibit the formation of biofilms and accelerate infected-wound healing. Moreover, it is well-known that Ag ions can combine with proteins, nucleic acid, and enzymes in microorganisms, causing a serious deformation of the bacteria membrane to ultimately kill bacteria 135. To this end, Cao *et al.* constructed an efficient and benign antibacterial depot (PDDA-Ag^+^-Cys-MoS_2_) with Cys-modified MoS_2_ loaded with minimum Ag ions and coated with cationic polyelectrolyte (**Figure 5B**) 136. This system displayed remarkable antibacterial activity toward both *E. coli* and *S. aureus* compared with an equivalent amount of silver nitrate, due to its enhancing accessibility of released Ag ions to the bacterial wall (**Figure 5C, D**). In addition, antibiotic-loaded MoS_2_ nanosheets and MoS_2_‑modified curcumin nanostructures could effectively release antibacterial agents against multidrug-resistant bacteria 127, 128. To enhance the antibacterial activity of this disinfection process, high concentrations of 2D NBG nanocarriers are necessary. However, due to the toxicity of metallic ions, it is important to regulate the concentration of metal ions in an acceptable range. Therefore, the effective removal of metal ions and 2D NBG from *in vivo* also requires a deeper investigation in the future.

### 2.5 Synergistic Antibacterial

In the previous sections, we have detailed introduced the typical antibacterial mechanisms of 2D NBG based on their intrinsic antibacterial properties. However, the complexity, diversity, and multidrug resistance of bacteria seriously impede the development potential of 2D NBG for disinfection. The antibacterial mechanisms mentioned above also suffer from their respective shortcomings, which limit their further antibacterial application. For example, the mechanism of physical contact destruction could only kill the bacteria attached on the surface of 2D NBG, and the long-term antibacterial efficiency could significantly decrease with the degradation of nanomaterials. For the oxidative stress antibacterial mechanism, the antibacterial effects mainly depend on the specific surface area, conductivity, and size of the 2D NBG. Although light-induced antibacterial mechanisms were deemed as one of the generally accepted efficient antibacterial strategies, the targeted killing bacteria ability, the hypoxia infected areas, and the photocatalytic efficiency are still prominent factors limited their antibacterial performances. In addition, for the release of metallic ions or drugs, the overuse may also bring serious toxicity to normal tissues. Thus, current study trend has gradually converted from the concentration on mono-mode antibacterial to 2D NBG-mediated multi-mode synergistic antibacterial for enhanced antibacterial effect. Currently, 2D NBG was often employed as nanocarriers to deliver other antibacterial agents (such as drugs, metallic ions, gas donor, and antibacterial polymers) into infected areas, which attain striking synergistic antibacterial effects that are stronger than that of any antibacterial strategy alone. Here, we summarize several common 2D NBG-mediated synergistic antibacterial methods: (i) synergy of PTT with PDT 137-139; (ii) synergy of PTT with metallic ions release (Ag, Au, *etc.*); (iii) synergy of chemotherapy with PTT 140; (iv) synergy of PTT with oxidative stress 79; (v) synergy of PTT with catalytic 139, 141-144; (vi) and tri-modal synergistic antibacterial 145, 146. At the same time, the synergetic antibacterial mechanism and application are detailedly discussed.

**Figure 6:**
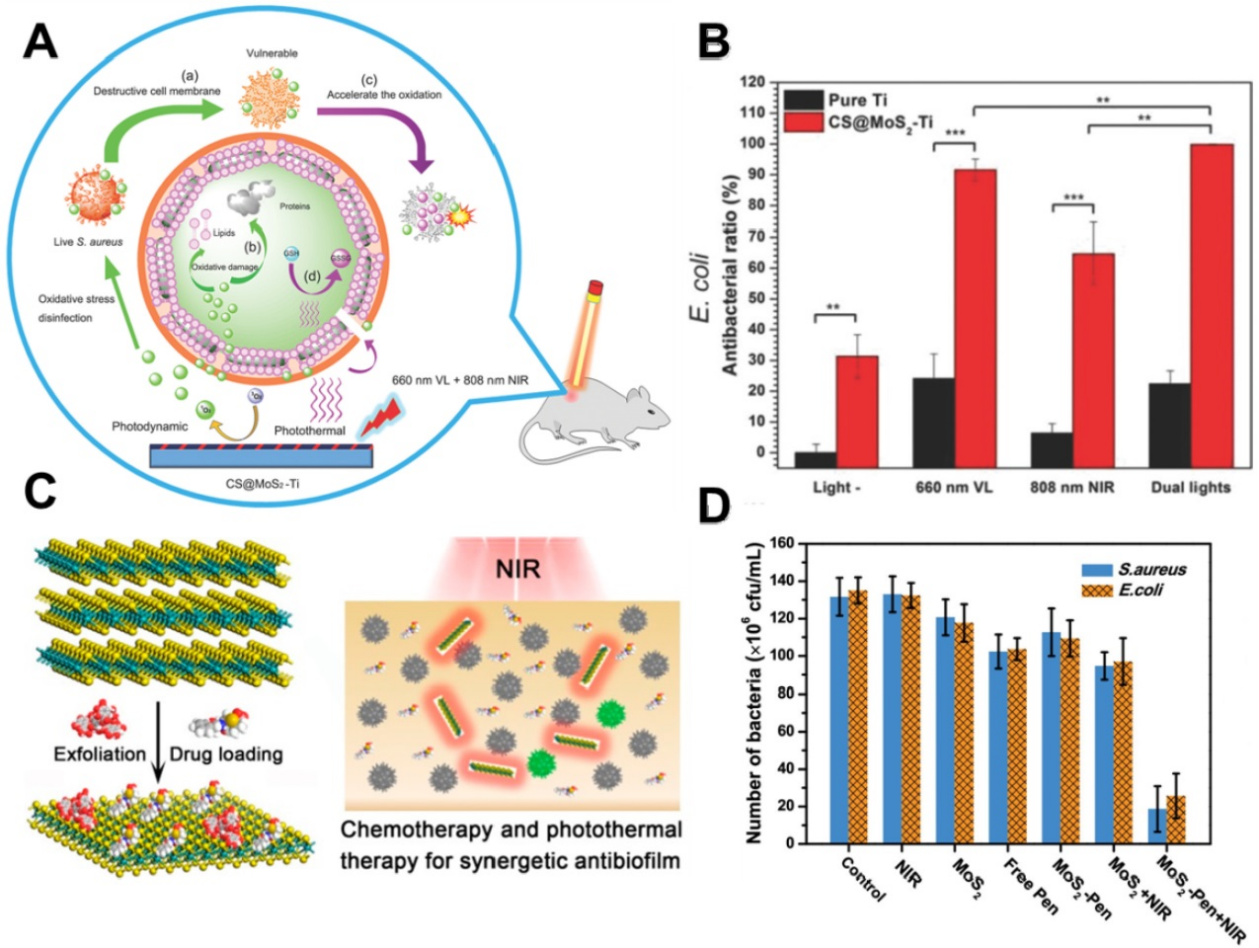
(**A**) Schematic illustration of CS@MoS_2_ hybrid coating constructed on the surface of Ti material for synergistic photodynamic and photothermal antibacterial action under the dual lights irradiation. (**B**) The antibacterial activity *E. coli* and *S. aureus* of pure Ti and CS@MoS_2_-Ti under the conditions of darkness (light) and after exposing to 660 nm visible light; 808 nm NIR; dual lights for 10 min. Reproduced with permission from 137, copyright 2018 WILEY-VCH Verlag GmbH & Co. KGaA, Weinheim. (**C**) The Pen-loaded MoS_2_ nanosheets exhibit outstanding antibiofilm activity *via* synergetic chemotherapy and photothermal therapy. (**D**) Number of *S. aureus* and *E. coil* in the presence of different antibacterial agents: control, NIR light, MoS_2_ nanosheets, free Pen, MoS_2_-Pen nanosheets, MoS_2_ nanosheets with NIR light, and MoS_2_-Pen nanosheets with NIR light. Reproduced with permission from 140, copyright 2018 American Chemical Society.

**Figure 7:**
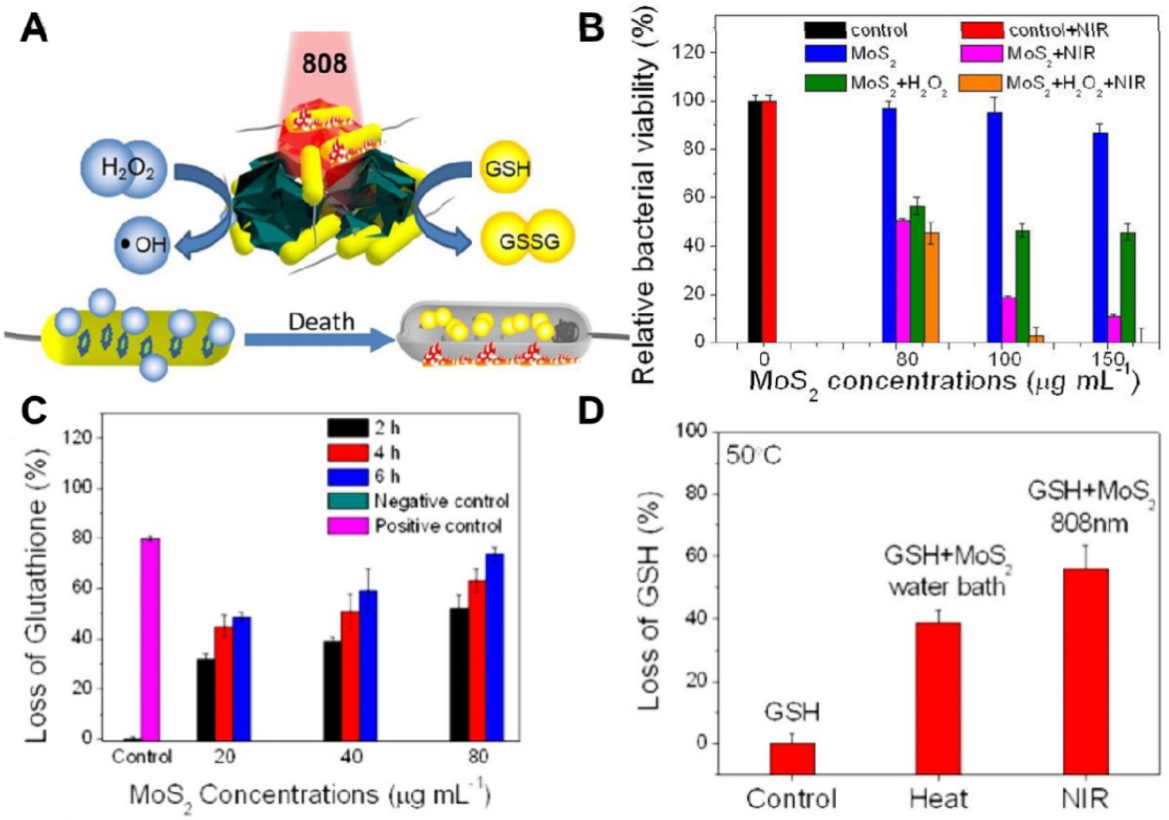
(**A**) Schematic illustration of PEG-MoS_2_ for peroxidase catalyst and photothermal synergistic antibacterial. (**B**) Relative bacterial viability of *Ampr E. coli* incubated with different concentrations PEG-MoS_2_ NFs with or without H_2_O_2_ (100 μM) under 808 nm laser irradiation. (**C**) Loss of GSH plot after incubation with PEG-MoS_2_ at different time intervals. (**D**) Loss of GSH plots heated by water bath and NIR 808 nm irradiation, respectively, for 20 min at 50°C. Reproduced with permission from 143, copyright 2018 American Chemical Society.

In recent years, PTT antibacterial has been widely accepted by researchers. The generation of heat by PTT can not only directly destroy the bacteria but also accelerate blood flow so as to effectively improve the intracellular generation of ROS for enhanced PDT. To this end, Wu's group synthesized a chitosan-assisted MoS_2_ (CS@MoS_2_) hybrid with a coating on the surface of Ti material, which showed rapid *in situ* antibacterial effect 147. They demonstrated that the synergistic effects of PDT and PTT actions of CS@ MoS_2_-Ti under dual lights irradiation (660 nm visible light and 808 nm NIR) could significantly enhance the antibacterial effect towards *E. coli* and *S. aureus* (**Figure 6A, B**). Besides, Wu's group prepared hybrid nanosheets based on g-C_3_N_4_-Zn^2+^@graphene oxide for rapid sterilization and accelerated wound healing under dual light irradiation 139. They found that the hybrid nanosheets could achieve an antibacterial ratio over 99.1% within a short time due to the synergistic effects of PDT and PTT. In addition, Wang's group used polydopamine (PDA), MoS_2_ nanosheets and silver nanoparticles to form MoS_2_@PDA-Ag nanosheets (MPA) as a novel dual antibacterial nanoagent 148. On the one hand, under NIR irradiation, the MPA nanosheets could generate heat, and directly damage the bacterial biofilm and the bacterial membrane. On the other hand, PTT also accelerates Ag ions release, enhancing the antibacterial efficacy of Ag ions. Therefore, the therapy strategy of combining PTT and metallic ions release is helpful for addressing the lower single antibacterial effect. In addition, Yuan *et al.* fabricated a functional MoS_2_/PDA-arginine-glycine-aspartic acid (RGD) to inhibit bacterial infection *in situ* and improve osseointegration 79. They found that NIR-induced hyperthermia of MoS_2_/PDA-RGD samples could efficiently increase GSH oxidation. Besides, the intrinsic ROS-independent oxidative stress of MoS_2_ nanosheets could also destroy the integrity of the bacterial membrane, finally significantly killing bacteria. After NIR irradiation, the antibacterial efficiency of MoS_2_/PDA-RGD reached 96.6% for *S. aureus* and 97.8% for *E. coli*, which is ascribed to the synergism between PTT and oxidative stress. Some antibiotic drugs, such as penicillin (pen), curcumin and lysozyme can be controllably released from 2D NBG surface. Based on which, Zhang *et al.* constructed an antibacterial nanomaterial through a polyphenol-assisted exfoliation strategy to exfoliate MoS_2_ nanosheets and loading antibiotic drugs pen onto MoS_2_ nanosheets surface (**Figure 6C**), which exhibited robust antibacterial effect *via* a synergetic of NIR-driven PTT and Pen-induced chemotherapy (**Figure 6D**) 140. Furthermore, our previous study demonstrated that polyethylene glycol functionalized MoS_2_ nanoflowers (PEG-MoS_2_ NFs) with peroxidase-like catalytic activity could be used for treating *in vivo* wound infection, which could convert low dosage clinical H_2_O_2_ into more toxic •OH. Together with the NIR photothermal ability of MoS_2_, it could realize synergetic PTT/catalytic antibacterial (**Figure 7A**, B) 143. Intriguingly, PEG-MoS_2_ NFs exhibited a time-dependent and hyperthermia-enhanced oxidation behavior, and after incubation with PEG-MoS_2_ NFs (80 μg mL^-1^) for 6 h the statistical loss of GSH can reach to 73.4% (**Figure 7C**). Furthermore, compared to the water bath group at 50 °C, the hyperthermia induced by PEG-MoS_2_ NFs showed a much higher GSH oxidation level (**Figure 7D**). Recently, Qu's groups firstly developed a nanozyme with abundant defects that have enhancing bacterial capturing abilities and efficient enzyme catalysis antibacterial effects 144. Briefly, the nanozyme with rough surface not only can efficiently trap bacteria, but also the defect-rich edges exhibit higher intrinsic peroxidase-like activity than pristine structures, which generate amounts of toxic •OH around the bacteria and exhibit considerable bacterial inhibition effect. Aside from the above mentioned dual-mode synergistic antibacterial, Yin *et al.* designed a tri-modal synergistic antibacterial agent (MoO_3-x_-Ag) for cleaning the bacterial contaminated water environment 145. They found that the as-designed MoO_3-x_-Ag killed pathogenic bacteria mainly through (i) photothermal effect of MoO_3-x_ nanosheets upon NIR irradiation; (ii) NIR light excitation of MoO_3-x_-Ag trigger the release of Ag ions; and (iii) photocatalytic reaction. Moreover, Roy *et al.* prepared chitosan exfoliated MoS_2_ nanosheets (CS-MoS_2_) and investigated their intrinsic antibacterial mechanism 146. They found that the CS-MoS_2_ nanosheets could kill bacteria through a synergistic effect of ROS-dependent oxidative stress, physical contact caused membrane damage and metabolic inactivation.

### 2.6 Effect of Physicochemical Properties on the Antibacterial of 2D NBG

It is well-known that the antibacterial effect of 2D nanomaterials can be significantly influenced by their intrinsic physicochemical properties, such as size, shape, number of layers, phase structure, and surface functionalization. Firstly, the size of 2D nanomaterials is a key factor in governing the antibacterial effect because size can strongly influence the dispersibility and adsorption ability, which are crucial to the interactions between 2D nanomaterials and bacteria. For instance, a relatively smaller size of Ag nanoparticles can increase the interaction with bacteria and exhibit higher antibacterial activity 149-151. Then, the shapes of 2D nanomaterials are also found to largely influence the germicidal ability.

It was revealed that the shapes of 2D nanomaterials would affect its interaction strength with the bacterial membrane, which greatly influences electron-transfer-based oxidative stress antibacterial activities. For example, Ananth *et al.* prepared spherical and sheet-like ruthenium oxide (RuO_2_) nanomaterials, and their shape-dependent anti-bacterial activities were carried out. Results showed that RuO_2_ nanosheets displayed higher antibacterial activity compared with RuO_2_ nanospheres 152. Moreover, the numbers of 2D NBG layers also influence antibacterial performance. Normally, thinner nanomaterials can easily damage the bacterial cell wall, and causing outstanding antibacterial effect. For instance, Sun *et al.* used N,N'-dimethylpropyleneurea as a new exfoliating solvent to effectively produce higher crystalline and free defects of BP nanosheets, and found that exfoliated BP nanosheets exhibited thickness-dependent photothermal antibacterial effects 153. In addition, the phase structure of layered TMDCs nanomaterial also affects their antibacterial activity. In general, due to the differences in coordination geometry between metal and chalcogen atoms, TMDCs mainly include the 1T phase and 2H phase 154. The 1T phase structure of TMDCs with metallic character can be used in cell destruction using NIR PTT effect 88, while the 2H phase structure with semiconducting and photoluminescence property can be utilized in visible light-induced water disinfection 97. According to research findings, the antibacterial capacity of exfoliated 1T-MoS_2_ nanosheets is higher than the annealed exfoliated 2H-MoS_2_ nanosheets, which is attributed to the higher electron conductivity in 1T-MoS_2_ nanosheets 75. Finally, the surface functionalization also affects the antibacterial performance of 2D NBG through changing their surface charge 155. For instance, 2D nanomaterials modified with positively charged groups could efficiently attach to the negatively charged bacterial membrane, which presents higher antibacterial activities 74.

## 3. 2D NBG Antibacterial Nanomaterials

In the past few years, there have been large amounts of research that concentrate on 2D NBG and their bactericidal applications. Taking inspiration from the excellent antibacterial performances of graphene, the potential antibacterial applications of 2D NBG, such as transition-metal dichalcogenides/oxides (TMDC/Os), metal-based nanocompounds, C_3_N_4_, BP, MXenes and other burgeoning 2D NBG materials have also been researched. Here, a summary of the recent progress of the 2D NBG antibacterial applications is in **Table 1**, and the detailed introduction is given below.

### 3.1 Transition-Metal Dichalcogenides/Oxides Antibacterial Nanomaterials

**Figure 8:**
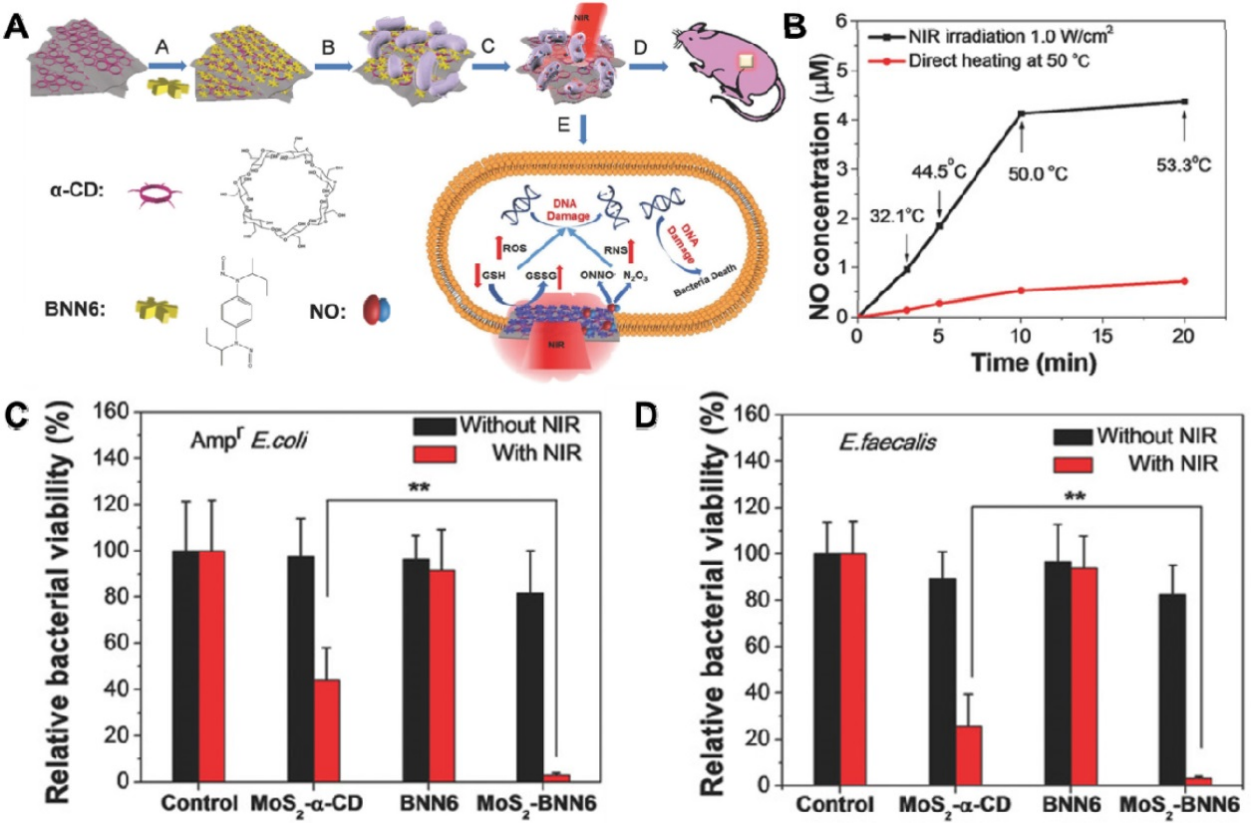
(**A**) Schematic illustration of MoS_2_-BNN6 as NIR laser-mediated NO release nanovehicle for synergistic eliminating bacteria. (**B**) Effects of direct heating and 808 nm laser irradiation on NO release from MoS_2_-BNN6. The corresponding bacterial viabilities of (**C**) Ampr *E. coli*, **D**) *E. faecalis* treated with PBS, MoS_2_-α-CD, BNN6, and MoS_2_-BNN6 without or with 808 nm laser irradiation (1.0 W cm^-2^, 10 min). Reproduced with permission from 157, copyright 2018 WILEY-VCH Verlag GmbH & Co. KGaA, Weinheim.

In the last few decades, 2D TMDC/Os with general chemical formula of AB_X_ (A: transition-metal, B: chalcogen), have been studied extensively in nanotechnology field owing to their unique graphene-like properties, which are composed of a “sandwich” structure of “B-A-B” or “B-A-O” through weak Van der Waals forces 178-180. Prototypical TMDC/Os, MoS_2_, MoO_2_, MoSe_2_, WO_3-x_, and WS_2_ are the most extensively explored ones 181, 182. For example, MoS_2_ nanosheets have exhibited great potential in the biomedical field due to their unique properties correlated with their 2D ultrathin atomic layer structure and high surface area 183. Specifically, owing to its layer-dependent bandgap, MoS_2_ nanosheets with very broad photodetection ability can be used as a candidate for photothermal/photocatalytic antibacterial 183, 184. Taking advantage of this feature, our groups fabricated a biocompatible 808 nm laser-mediated NO-releasing MoS_2_-BNN6 nanovehicles for low-cost, rapid, and effective antibacterial (**Figure 8A**) 157. We demonstrated that MoS_2_ nanosheets with high photothermal conversion efficiency and hyperthermia could control NO delivery and release upon 808 nm laser irradiation (**Figure 8B**). The synergistic effect of PTT and NO release lead to outstanding germicidal ability toward *Amp^r^ E. coli* (**Figure 8C**) and *E. faecalis* (**Figure 8D**). Moreover, comet assay results proved that the MoS_2_-BNN6 nanovehicles presented with PTT/NO synergetic antibacterial activities could cause great DNA damage. The *in vivo* wound healing in mice demonstrated the practical applicability of this antibacterial strategy. Moreover, Liu *et al.* fabricated few-layered vertically aligned MoS_2_ (FLV-MoS_2_) films with the ability to harvest the whole spectrum of visible light for effective photocatalytic water disinfection 97. Due to the increased bandgap of MoS_2_, from 1.3 eV (bulk material) to 1.55 eV (FLV-MoS_2_), FLV-MoS_2_ could generate considerable ROS for bacteria inactivation in water under visible light irradiation. They also found that the additional deposition of Cu or Au onto FLV-MoS_2_ films could assist electron-hole pair separation and catalyze ROS production reactions, which allowed FLV-MoS_2_ to realize a rapid inactivation of >99.999% bacteria within only 20 min. Similarly, Cheng *et al.* constructed a pyramid-type MoS_2_ (pyramid MoS_2_) on transparent glass using the chemical vapor deposition method for highly effective water disinfection under visible light irradiation 158. They also found that the pyramid MoS_2_ has a smaller bandgap, which could harvest a wide spectrum of sunlight and generate more ROS for killing bacteria.

Aside from the photo-induced germicidal ability, the peroxidase-like activity of MoS_2_/MoSe_2_ nanosheets has also been verified. Our group constructed functionalized nano-MoS_2_ with peroxidase catalytic and NIR PTT for synergetic antibacterial 143. Besides, the imbalance of bacterial flora may increase resistance, which demands the development of strain-selective bactericidal strategies. To this end, Qu's groups designed an intelligent photo-responsive Gram- selective antibacterial system citraconic anhydride-modified MoS_2_ 185. This nanosystem possesses charge-selective antibacterial potential because the surface charge could be modulated by altering light irradiation time, and the simultaneous lower pH value would activate the peroxidase-like activity of MoS_2_ nanozymes as well as increase antibacterial effects. In addition, Huang *et al.* used carboxyl-modified silk fibroin as the exfoliating agent to high-yield synthesize thin-layer MoSe_2_ nanosheets, and revealed that the superior peroxidase-like activity of MoSe_2_ nanosheets could effectively resist bacterial infections and promote wound healing 159. Furthermore, the antibacterial activity of WX_2_ nanosheets has also been valued in recent years 186. Bang *et al.* designed a facile and effective exfoliation technique to fabricate WX_2_ (X=S or Se) nanosheets by using single-stranded DNA (ssDNA) of high ssDNA molecular weight, and investigated the antibacterial activity of the as-prepared WX_2_. They found that WSe_2_-ssDNA nanosheets had remarkable antibacterial activity due to their strong GSH oxidation capacity, which suggested the ROS-independent oxidative stress antibacterial properties of WSe_2_-ssDNA nanosheets 65, 85.

### 3.2 Metal-Based 2D Antibacterial Nanomaterials

**Figure 9:**
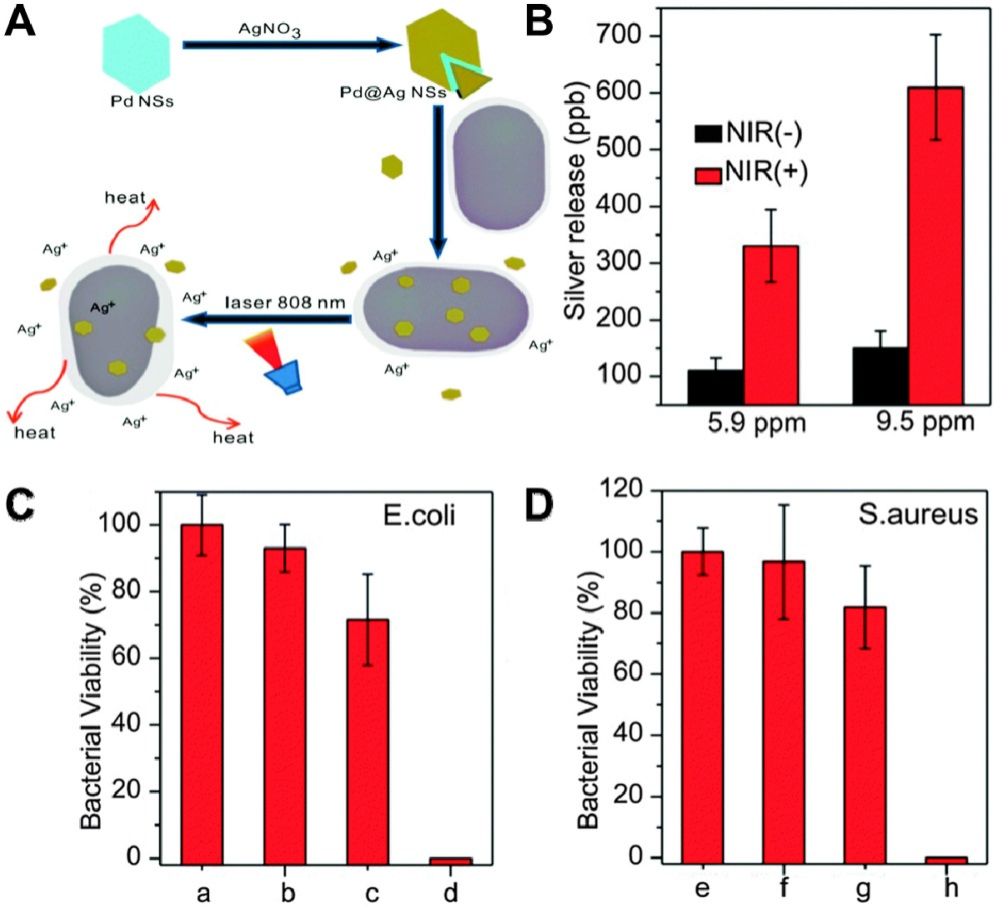
(**A**) Schematic illustration of the Pd@Ag nanosheets for synergetic treatment of bacteria. (**B**) Silver released from 5.9 and 9.5 ppm of Pd@Ag nanosheets with and without light irradiation. Histograms of (**C**) *E. coli* and (**D**) *S. aureus* viability treated with Pd@Ag nanosheets under NIR irradiation. Reproduced with permission from 162, copyright 2015 The Royal Society of Chemistry.

It has been demonstrated that metallic nanoparticles, including Ag, Au 187, Ti, Cu 188, Zn 189, Mg 190-192 and Ni 192, exhibit potential antibacterial activity. However, compared to 2D nanomaterials, these metallic nanoparticles show compromised bactericidal efficiency due to its small specific surface area and fewer surface-active sites. Therefore, the strategy to overcome the shortcomings and obtain high-performance antibacterial effect of metal alone is either to decorate 2D nanosheets with metallic nanoparticles or prepare 2D metallic nanosheets 193-195. Towards this direction, Mo *et al.* prepared Pd@Ag nanosheets through the reduction of Ag ions with formaldehyde on the surface of Pd nanosheets. It could realize a synergetic antibacterial effect of PTT and Ag ions released under the irradiation of NIR light (**Figure 9A**) 162. The amounts of released Ag from Pd@Ag nanosheets were monitored by inductively coupled plasma mass spectrometry (ICP-MS), and results showed that NIR irradiation could significantly increase silver release compared to non-irradiated controls (**Figure 9B**). Interestingly, the bacteria viability indicated that NIR laser or Pd@Ag nanosheets alone did not kill bacteria effectively, while Pd@Ag nanosheets upon NIR irradiation could realize efficient bacteria-killing effect as almost 100% bacteria was killed after the synergistic treatment for 10 min (**Figure 9C, D**). Therefore, Pd@Ag nanosheets with outstanding photothermal conversion effect could not only generate heat to kill bacteria, but also destroy the bacterial membrane with released Ag ions under the irradiation of NIR light. Recently, 2D nanosized metallic oxides, such as TiO_2_ 196, CuO 188, and ZnO 197, 198, have shown excellent antibacterial performance and drawn widespread attention due to their favorable photocatalytic activities, benign biosafety, strong oxidizing power, and rich surface-active sites. Among them, ultrathin TiO_2_ nanosheets are confirmed as the most outstanding antibacterial, which is attributed to their numerous advantages, such as low-cost, low-toxicity, good stability, as well as robust oxidizing capability. In general, TiO_2_ nanosheets have three phase structure: brookite, rutile and anatase. Among them, anatase TiO_2_ nanosheets have been widely applied as a photocatalyst 196. To explore the in-depth capacity of TiO_2_, Arnab *et al.* prepared MoS_2_-TiO_2_ nanocomposites with significant antibacterial ability 165. In addition, Ma *et al.* fabricated ultrathin Fe_3_O_4_-TiO_2_ nanosheets (Fe_3_O_4_-TNS) using lamellar reverse micelles and solvothermal method for efficient antibacterial application 199. On the one hand, Fe_3_O_4_-TNS could effectively capture photo-induced electron and accelerate the separation of electron-hole pairs. On the other hand, Fe_3_O_4_-TNS showed superior antibacterial activity to *E. coli* under the irradiation of solar light. Moreover, Wang *et al.* designed a bifunctional layered Cu_2_S nanoflowers/biopolymer-incorporated electrospun membrane for tumor therapy and as a source of Cu ions for wound healing 89. Despite the tremendous antibacterial potential of metal-based nanomaterial shown from these studies, it is important to investigate their cytotoxicity and immune response effect on human cells. Therefore, metal-based antibacterial nanomaterials deserve further research regarding their *in vivo* biological effect before clinical sterilization application.

### 3.3 Nitride-Based Antibacterial Nanomaterials

**Figure 10:**
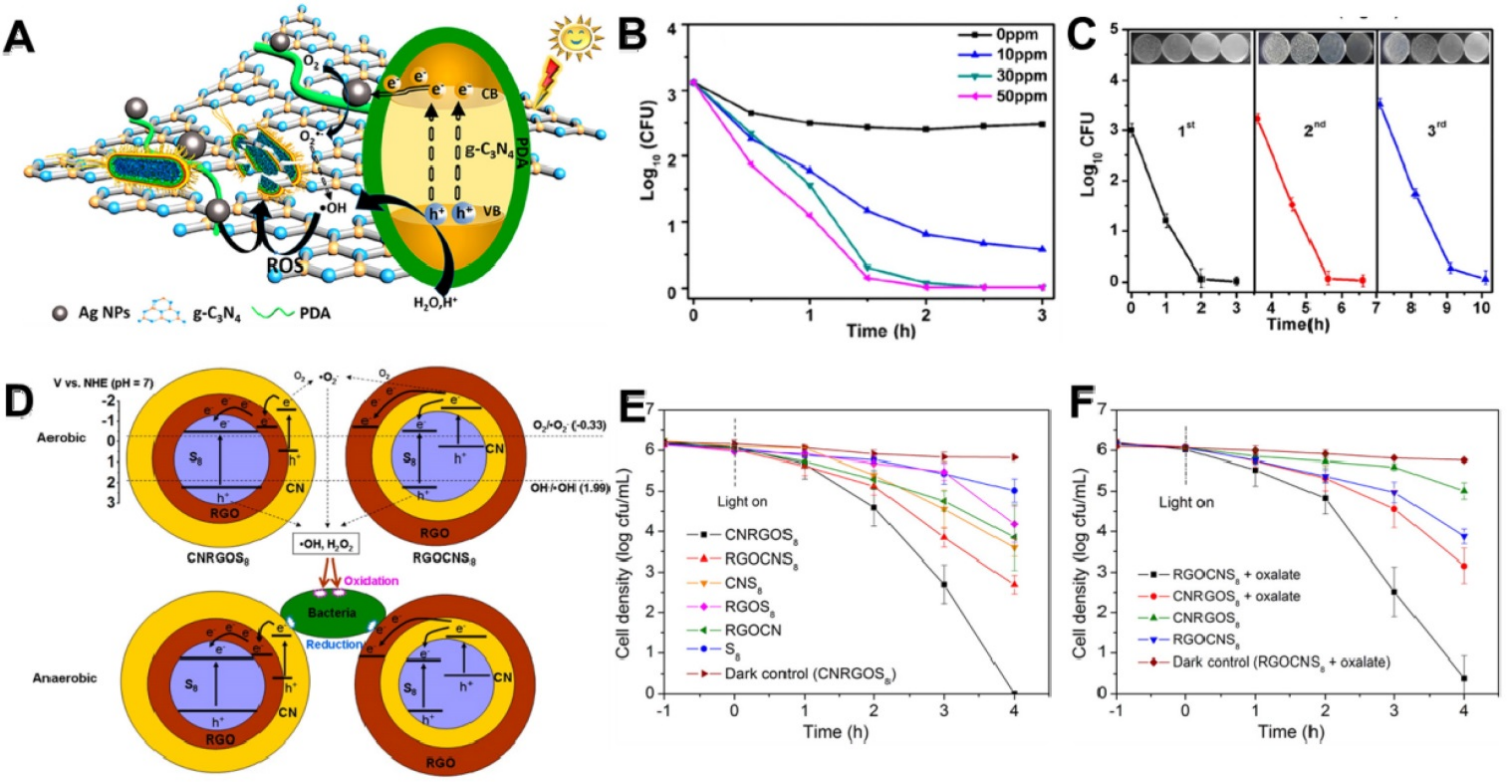
(**A**) Bactericidal mechanism for Ag/PDA/g-C_3_N_4_ bio-photocatalyst. (**B**) Bactericidal time curve profiles of *E. coli* treated with different concentrations of Ag/PDA/g-C_3_N_4_ (1:2) within 3 h. (**C**) Three-cycle run experiments of photocatalytic disinfection of 30 ppm Ag/PDA/g-C_3_N_4_ (1:2) under visible-light irradiation. Reproduced with permission from 170, copyright 2018 American Chemical Society. (**D**) Schematic illustration of the VLD photocatalytic bacterial inactivation mechanisms of CNRGOS_8_/RGOCNS_8_ in aerobic condition, and CNRGOS_8_/RGOCNS_8_ in anaerobic condition. Photocatalytic inactivation efficiency against *E. coli K-12* without (**E**) and with (**F**) 0.5 mmol/L sodium oxalate in the presence of the samples (100 mg/L) in anaerobic condition under visible light irradiation. Reproduced with permission from 113, copyright 2018 American Chemical Society.

C_3_N_4_ was first synthesized by Berzelius in the 1830s, subsequently Liebig named it “melon” 111. Recently, g-C_3_N_4_ nanosheets, a burgeoning nitride-based polymeric semiconductor material with laminar structure and narrow bandgap, have attracted giant attention on the antibacterial field owing to their highly efficient photocatalytic performance, large surface area, good chemical stability and adjustable electron band structure 200. Wu *et al.* once synthesized an Ag/polydopamine/g-C_3_N_4_ bio-photocatalyst antibacterial agent using *in situ* reduction method (**Figure 10A**) 170. On the one hand, the photocatalytic activity of Ag/polydopamine/g-C_3_N_4_ was improved through Ag NPs and polydopamine under the irradiation of visible light, followed by the generation of abundant ROS (particularly •OH). On the other hand, light could accelerate the release of Ag ions. Finally, the Ag/polydopamine/g-C_3_N_4_ displayed excellent antibacterial activity by a synergistic effect between Ag ions and PCT of g-C_3_N_4_ nanosheets (**Figure 10B**). Simultaneously, this bio-photocatalyst has commendable biocompatibility and outstanding stable antibacterial activity (**Figure 10C**). To further advance the use of g-C_3_N_4_ as photocatalysts, Wang *et al.* firstly designed a novel metal-free heterojunction through coating cyclooctasulfur (*α*-S_8_) with rGO and g-C_3_N_4_ nanosheets for photocatalytic disinfection under visible light 113. They found that the photocatalytic bacteria inactivation mechanism of this nano-heterojunction mainly through photogenerated ROS in an aerobic environment or through electron transfer induced oxidative stress under anaerobic conditions (**Figure 10D**). Intriguingly, the results showed that a S_8_ (core)/rGO (inner shell)/g-C_3_N_4_ (outer shell) (CNRGOS_8_) arrangement exhibited higher antibacterial effect under aerobic conditions (**Figure 10E**), whereas a S_8_ (core)/g-C_3_N_4_ (inner shell)/rGO (outer shell) (RGOCNS_8_) arrangement showed excellent antibacterial ability under anaerobic conditions (**Figure 10F**). In addition, Li and co-workers fabricated g-C_3_N_4_ nanosheets functionalized hydrophilic composite membranes, and found that the antibacterial ability largely increased with the modification of g-C_3_N_4_ nanosheets 166. Furthermore, Wang *et al.* reported that vanadate quantum dots-inset g-C_3_N_4_ (vanadate QDs/g-C_3_N_4_) nanosheets showed faster photocatalytic disinfection with abundant ROS compared to bare g-C_3_N_4_ nanosheets 171. Moreover, to reduce the toxicity risk of heavy metal species, Chen's groups created a novel all-organic self-assembled semiconductor photocatalytic nanomaterial C_3_N_4_/PDINH heterostructure for photocatalytic antibacterial by recrystallization of PDINH (perylene-3,4,9,10-tetracarboxylic diimide) on the surface of C_3_N_4_ nanosheets* in situ* 201. Intriguingly, they found that the absorption spectrum of heterostructure were greatly extended from UV to NIR, enhancing the photocatalytic effect to generate more ROS for better bactericidal, while revealing low-toxicity to healthy tissue cells. Simultaneously, the C_3_N_4_/PDINH heterostructure could quickly cure the* in vivo* infected area, and promote wound regeneration. Taken together, although C_3_N_4_-based photocatalyst has reported in various biomedical fields, its photocatalytic efficiency is still relatively low to meet the requirements of practical applications. Therefore, enhancing the catalytic efficiency of C_3_N_4_ nanosheets in the future is highly expected.

### 3.4 Black Phosphorus-Based Antibacterial Nanomaterials

**Figure 11:**
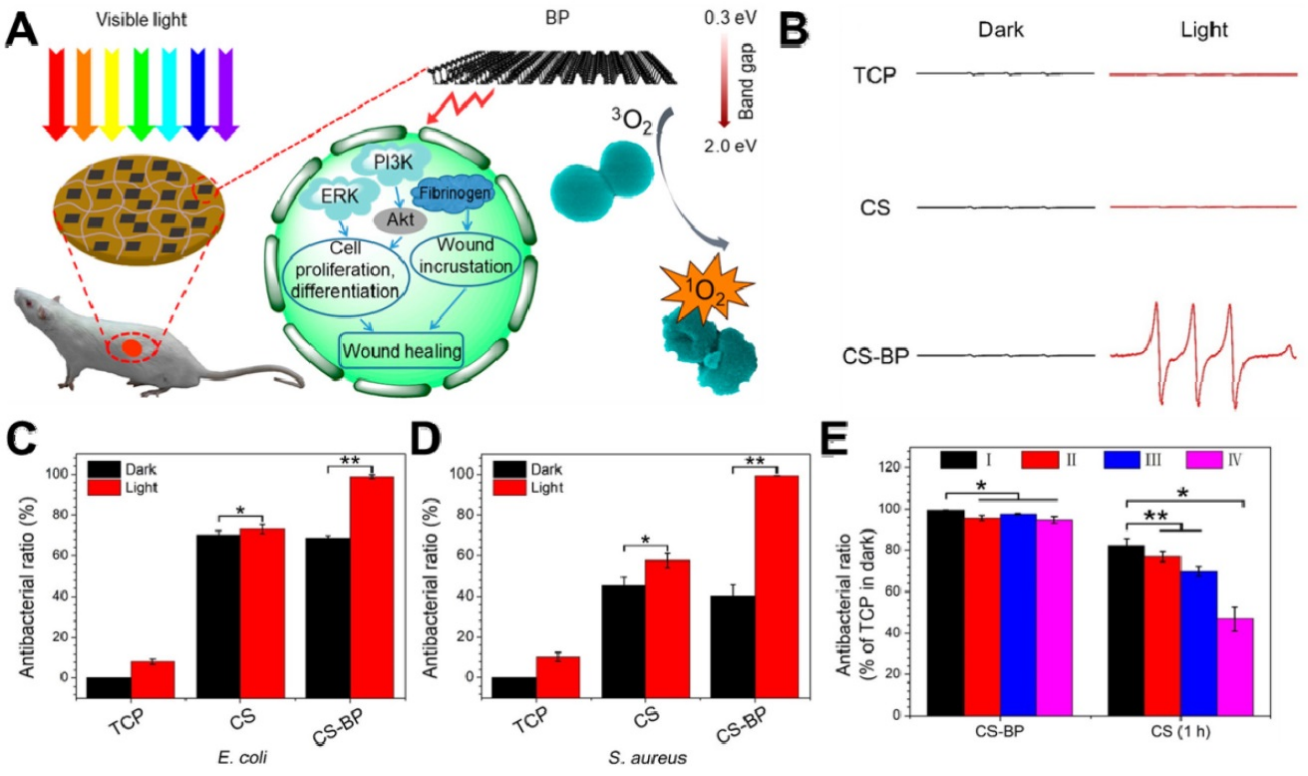
(**A**) Sterilization under visible light irradiation and the process of stimulating skin cell behaviors that can promote the regenerative activities of the skin cells and actively participate in skin regeneration to accelerate bacteria-accompanied wound healing using BP-based hydrogel. (**B**) Electron spin resonance spectra. The corresponding abilities of the samples (TCP, the CS hydrogel, and the CS-BP hydrogel) to kill *E. coli* (**C**) and *S. aureus* (**D**); (**E**) Corresponding abilities of the samples to kill *S. aureus*; the CS-BP hydrogel and CS hydrogel were repeatedly challenged with *S. aureus* under simulated sunlight for 10 min and in the darkness for 1 h, respectively, repeatedly up to four times. Reproduced with permission from 39, copyright 2018 American Chemical Society.

BP, a rising star of 2D nanomaterials family, has attracted great attention since 2014 202, 203. Recently, 2D BP nanosheets have attracted widespread attention due to their fascinating thermal/optical/electrical performance, and the weak Van der Waals forces allow its easy exfoliation into ultrathin 2D nanosheets 204, 205. Owing to their metal-free semiconductor features and thickness-dependent band gap, BP nanosheets possess a very broad light absorption across entire visible regions 206. Therefore, 2D layered BP nanosheets can be used to produce ^1^O_2_ under visible light, which can be applied to PDT 207. Towards this direction, Mao *et al.* fabricated a BP-based hybrid hydrogel (CS-BP) therapeutic system for repeatable PDT disinfection and promote wound healing (**Figure 11A**) 39. They used electron spin resonance (ESR) spectra to trap ^1^O_2_, and found that only CS-BP hydrogel could rapidly generate ^1^O_2_ under visible light irradiation (**Figure 11B**). Meanwhile, under light irradiation with simulated sunlight, CS hydrogel showed moderate bactericidal efficacy while CS-BP could kill most bacteria (*E. coli* and *S. aureus*) within 10 min (**Figure 11C, D**). Moreover, the CS-BP hydrogel still maintain favorable reusable antibacterial efficacy even after four repeatedly challenge with high concentrations of *S. aureus* (**Figure 11E**). In addition, Huang *et al.* prepared thin-layer BP@ silk fibroin nanosheets with subtle solution-processability using silk fibroin as an exfoliating agent, and revealed that BP@ silk fibroin nanosheets not only could be fabricated into various formats but also show excellent PTT disinfection and wound repair ability 92. Based on the above attractive findings, BP-based nanomaterials hold great promise for material science, biomedicine, and biotechnology in the future.

### 3.5 MXenes Antibacterial Nanomaterials

**Figure 12:**
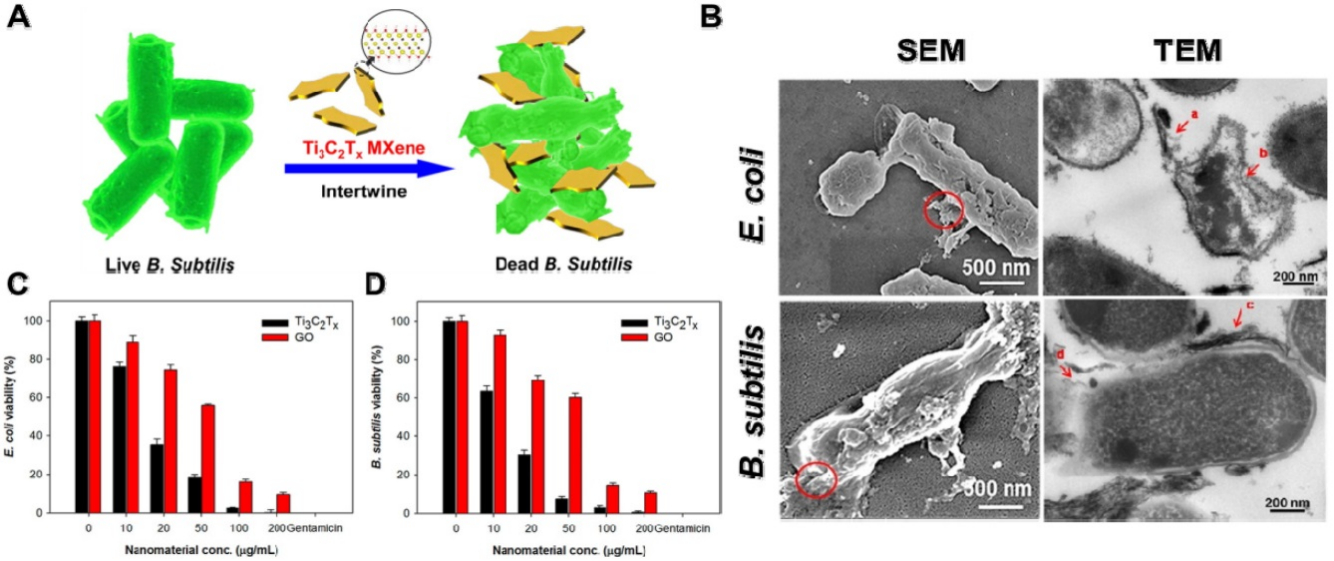
(**A**) Schematic illustration of the interaction between Ti_3_C_2_T_x_ and bacteria. (**B**) SEM and TEM images of *E. coli* and *B. subtilis* cells treated with Ti_3_C_2_T_x_ MXenes. Cell viability measurements of **C**) *E. coli* and **D**) *B. subtilis* treated with an aqueous suspension of Ti_3_C_2_T_x_ MXenes. Reproduced with permission from 173, copyright 2016 American Chemical Society.

MXenes are a very new member of 2D transition metal carbides/nitrides with a general formula of M_n+1_A_n_T_x_ (M stands for transition metal, A is carbon or nitrogen, and T is surface-terminating functional groups, such as -OH, -O, or -F) 25, 208. So far, approximate 70 kinds of MXenes have been reported, among which Ti_3_C_2_T_x_ is the most representative MXenes material 209. Recently, studies demonstrated that Ti_3_C_2_T_x_ MXenes nanosheets display outstanding antibacterial performances owing to their ultrathin lamellar morphology and unique physiochemical properties 209-211. For instance, Rasool *et al.* synthesized Ti_3_C_2_T_x_ MXenes colloidal suspension using ultrasonication in argon (Ar) gas and first demonstrated their outstanding antibacterial behavior towards *E. coli* and *B. subtilis* (**Figure 12A**) 173, 212. In comparison to GO, the Ti_3_C_2_T_x_ MXenes nanosheets displayed distinct dose-dependent bactericidal efficacy, and up to 98% of bacterial were killed after incubated with 200 μg/mL Ti_3_C_2_T_x_ for 4 h (**Figure 12C, D**). According to the SEM and TEM images, they proposed that the antibacterial mechanism of Ti_3_C_2_T_x_ MXenes nanosheets were ascribed to the synergism of sharp edge induced membrane damage and electron transfer rendered oxidative stress (**Figure 12B**). Then, they further fabricated Ti_3_C_2_T_x_ MXenes nanosheets coated with polyvinylidene fluoride (PVDF) membranes by vacuum-assisted filtration method and further studied their antibacterial ability towards *E. coli* and *B. subtilis* 174. Compared to fresh Ti_3_C_2_T_x_/PVDF membranes, the aged Ti_3_C_2_T_x_/PVDF membranes were more active in enhancing the overall antibacterial properties owing to the synergistic effect between Ti_3_C_2_T_x_ nanosheets and the formation of anatase TiO_2_ nanocrystals with sharp edges. Moreover, Shamsabadi *et al.* reported that colloidal Ti_3_C_2_T_x_ MXenes nanosheets showed both size- and exposure time-dependent antibacterial properties. They found that the direct interactions between sharp edges of Ti_3_C_2_T_x_ MXenes nanosheets and bacteria membrane cause serious damage to the bacterial membrane 212. Taking into consideration that MXenes' chemical behavior depends on the kind of transition metals (such as Mo, Nb, V) and surface-terminating functional groups, different physicochemical properties are closely relevant to antibacterial effects. Therefore, fine regulated ultrathin MXenes nanosheets will open a wide door for multiple and extensive application in antibacterial coatings and biomedical applications.

### 3.6 Others 2D NBG Antibacterial Nanomaterials

In addition to the above-mentioned 2D NBG antibacterial nanomaterials, some other new members of 2D nanomaterials, such as layered double hydroxides (LDHs) 213-216, laponite (Lap) 217, 218, hexagonal boron nitride (BN) 176, III_2_-VI_3_ compounds (In_2_Se_3_, Bi_2_Se_3_, Sb_2_Se_3_) 90, 177, 219, 220, and RuO_2_ have also been deemed as promising antibacterial agents. For example, Wang *et al.* prepared various morphology of lysozyme modified LDHs through tuning the ratio of cations, and found that bloom flower structure of LDHs not only could load more lysozyme but also adhere to more bacteria, which show excellent antibacterial activity and wound healing ability 175. Moreover, Ghadiri *et al.* fabricated a Laponite/mafenide/alginate (Lap/Maf/Alg) film with remarkable antibacterial effects and good wound healing applications 221. Furthermore, Zhu *et al.* synthesized large quantities of In_2_Se_3_ nanosheets using a solvent exfoliation method. Their results revealed that the In_2_Se_3_ nanosheets possess excellent photothermal performance, which causes significant antibacterial effect 177. Similarly, Miao *et al.* synthesized new atomically thin antimony Sb_2_Se_3_ nanosheets using a liquid exfoliation method, which could kill bacteria through physical contact destruction and short-time hyperthermia sterilization under laser irradiation 90. Therefore, various 2D antibacterial materials with excellent bacteria inhibition efficacy should gain more researchers' attention.

## 4. Conclusions and Future Perspectives

In recent years, the increasing of bacteria multidrug resistance to traditional antibiotics has greatly hampered the development of antibacterial applications. Fortunately, various emerging 2D NBG, which show promising potential and good opportunities in addressing antibacterial issues, have demonstrated their excellent ability to kill drug-resistant bacteria. In this review, we systematically summarize the research progress of versatile 2D NBG from their antibacterial mechanisms to materials classification, and the effect of unique physiochemical properties on their antibacterial applications. Similar to graphene, 2D NBG also has features such as high specific surface area, better stability, and good biosafety, which make them one of the most advanced, attractive, and promising antibacterial nano-agents. Currently, the existing antibacterial mechanisms of 2D NBG mainly include physical contact destruction, oxidative stress, photo-induced (photothermal, photocatalytic and photodynamic) heat/ROS production to damage cellular components, controlled drugs or metallic ions releasing, and multi-mode synergistic antibacterial. Among them, synergistic antibacterial is the most effective bactericidal method, and photo-induced antibacterial, especially photocatalytic antibacterial, has attracted far-ranging attention in recent years. Furthermore, we also discuss the effect of the physiochemical characteristic of 2D NBG on antibacterial effects. In addition, we have divided the 2D NBG into the following categories: TMDC/Os, Metal-based, Nitride-based, BP, MXenes and other 2D NBG antibacterial nanoagents. Although extensive research demonstrated that 2D NBG has excellent antibacterial activities, studies regarding the clinical translation of the 2D NBG are still rare. Therefore, in order to realize more practical application, it requires us to focus on several important issues in the future:

I) *The systematical evaluation of the biosafety of 2D NBG is all-important before being translated into clinical practice*. Currently, 2D NBG has been exhibited tremendous potential in antibacterial, water disinfection, wound healing, and other biomedical filed. However, biosafety consideration is a prerequisite for the clinical antibacterial translation of these 2D nanomaterials. Although some reports have been conducted to investigate the dosage, incubation time, and surface modification-related biosafety of 2D NBG, such research is still insufficient and remains controversial for the biosafety evaluation of 2D NBG 222. Most recent studies have demonstrated that 2D NBG presents good biocompatibility and low toxic effects after suitable functionalization, however, their long-term biological effects* in vivo* still unexplored 223, 224. Firstly, although metal or metallic ions possess apparent antibacterial properties, studies of exploring and reducing their *in vivo* long-term toxicity are needed. Secondly, to accelerate the metabolism of 2D NBG, we can improve their biodegradability through surface functionalization or design ultrasmall size of 2D NBG to make them more easily cleared out of the body 225. Thirdly, developing 2D NBG with targeting antibacterial ability could not only specifically target bacteria to improve antibacterial efficiency but also reduce their toxicity and side effects to normal tissues. Finally, the mechanism for clearance pathways and other biological effects of 2D nanomaterials are still not clear. Thus, putting all these unresolved biosafety issues in the first place for deep investigation is essential, which is beneficial for their future preclinical or clinical antibacterial application.

II) *The deeper exploration of the interactions between 2D NBG-bacteria interfaces and related antibacterial mechanisms*. To date, lots of antibacterial mechanisms of 2D NBG have been widely recognized, but most of the mechanism studies are far from comprehensive. Thus, it is essential to deeply understand the antibacterial mechanisms and explore the interactions between nanomaterials and intracellular components of microorganisms.

III) *The optimization of the physicochemical properties of 2D NBG nanomaterials for enhancing antibacterial effects*. As mentioned above, size, shape, phase structure, number of layers and surface functional modification, all of them affect antibacterial activities. For example, different size of MoS_2_ nanosheets possesses different drugs load capacity, leading to different antibacterial activities. Moreover, the shape-dependent antibacterial effects of VS_2_ nanosheets were also revealed. Therefore, the systematic study on unique physicochemical properties relevant to the antibacterial effect should be carried out to gain the satisfying antibacterial efficiency of 2D NBG for better preclinical use.

IV) *The exploration of the optimal modality of synergistic antibacterial*. In this review, we have summarized various combined modalities for synergistic bactericidal. However, no reliable research has compared these synergistic therapeutic strategies together, causing some studies to randomly combine several treatment strategies to improve antibacterial efficacy. In addition, most of the current *in vivo* researches mainly focus on superficial wound healing, the infection in the deep site of tissue is still rarely studied. Furthermore, the ways of drug delivery also play an important role in improving the synergistic antibacterial effects. Therefore, to obtain the optimal synergistic modality of synergistic antibacterial, it is needed to compare existing synergistic antibacterial effect under the same experimental conditions or propose new synergy mechanisms of 2D NBG.

V) *The promotion of the clinical translation of 2D NBG fungicide*. Currently, various 2D NBG antibacterial nanoagents were studied by researchers, but too few clinic antibacterial agents were developed. Therefore, their clinic translation process still remains to be a giant challenge. Therefore, tremendous efforts are needed to facilitate the clinical translation of 2D NBG.

VI) The *optimization* of *antibacterial effects of 2D NBG by computational simulation*. Currently, with the explosive development of science and technology, computers can do exactly what humans cannot do through simulations. For example, Fan's group using coarse-grained molecular dynamics simulations studied the physical and mechanical interaction between lipid liposomes and hydrophobic nanosheets 226. They found that the size and the orientation of 2D nanomaterials affect the interaction between cells and nanomaterials, which is a good supplement of the experiment. Based on it, we can further study the unresolved antibacterial mechanisms by the combination of computational simulation and theoretical calculation.

Taken together, we believe that this review regarding 2D NBG antibacterials can not only deepen researchers' interest in the antibacterial field, but also provide a new insight in exploring the unknown bactericidal mechanism. We hope that more research can be carried out to develop new types of safe 2D NBG antibacterials for future clinical transformation.

## Figures and Tables

**Table 1:** A summary of 2D NBG for antibacterial application

Category	Nanomaterials	Morphology, size & surface modification	Type of Bacteria	Antibacterial Mechanism & effect	Refs.
**TMDC/Os**	CS@MoS_2_	Monolayer NSs, Chitosan modified	*E. coli, S. aureus*	PDT & PTT cause the disruption of membrane integrity & leakage of cytoplasmic components; >99%	41, 137, 156
	Magnetic MoS_2_	NSs, Chitosan functionalized	*E. coli., S. aureus*	Enhance the conjugation of bacterial & PTT; >90%	95
	MoS_2_-Pen	Monolayer NSs, 38.6± 1.3 nm, Pen-loaded	*E. coli., S. aureus*	chemotherapy & PTT synergetic effect; kill the majority of bacterial	140
	PEG-MoS_2_	334 nm NFs, PEG modified	*E. coli, B. subtilis*	Peroxidase-like catalysis and PTT synergetic antibacterial; up to 97%	143
	MoS_2_-BNN6	Single-layer NSs, 50~80 nm, α-cyclodextrin modified	*Amp^r^ E. coli, E. faecalis*	PTT & NO-enhanced free radical generation synergetic antibacterial, highly effective bacterial inactivation (>97.2%)	157
	pyramid MoS_2_@Ag	Triangles, 5 to 10 μm	*E. coli*	Photocatalytic generate ROS; more than 99.99%	158
	MoSe_2_	100 nm NSs, without modified	*E. coli, B. subtilis*	Peroxidase-like activity could catalyze H_2_O_2_ into •OH for antibacterial; ~100%	159
	MoO_3_	plate-like structures	*E. coli, S. aureus, E. faecalis, B. subtilis*	Disruption of the bacterial cell wall; Effective	63
	MoO_3-x_-Ag	Around 300 nm NSs, without modified	*E. coli, S. aureus*	PTT, Ag+ release & photocatalytic synergic effect; 99.2% of *E. coli* and 97.0% of *S. aureus* are killed	145
	WS_2_	2~3 layers 200 nm NSs	*E. coli, S. aureus*	ROS generating & damage the structural integrity of bacterial membrane	65
	WX_2_-ssDNA	65~650 nm NSs	*E. coli*	ROS-independent oxidative stress; around 82.3%	78
	PDMS/WS_2_	Single-layer less than 1 µm nanoflowers	*E. coli*	ROS generating; more than 99.99%	160
	WS_2_	Monolayer NSs, Ag decorated	*E. coli*	Ag enhance the photocatalytic efficiency; up to 96%	161
**Metal-based**	Pd@Ag	hexagonal plate-like shapes, 85 nm	*E. coli*, *S. aureus*	the synergic effect of PTT & NIR-triggered Ag^+^ release; almost 100% bacteria	162
	Ag/CS@MnO_2_-Ti	Chitosan functionalized	*E. coli*, *S. aureus*	PTT & Ag ions release synergic effect; up to 99.00%	163
	TiO_2_	Sizes controllable NSs, Amine modified	*E. coli*	Photocatalytic antibacterialactivity; around 89%	164
	MoS_2_-TiO_2_	Sheet-like morphology	*E. coli*, *S. aureus*	Effective	165
**Nitride-based**	g-C_3_N_4_	Plane structural, etching with HCl	*E. coli*	Photocatalytic & self-cleaning; reach 100%	166
	g-C_3_N_4_-Au	irregular nanosheets, about 200 nm, 1-pyrenebutanoic acid modified	*E. coli*	light-triggered ROS generation; Effective	167
	Bi_2_MoO_6_/g-C_3_N_4_	Plane structural	*E. coli*	PCT antibacterial; significantly effect	115
	Ag@g-C_3_N_4_	Ultrathin NSs, 20~40 nm	*E. coli*, *S. aureus & P. aeruginosa*	photocatalytic generated ROS; Effective	168, 169
	Ag/PDA/g-C_3_N_4_	PDA modified	*E. coli*	Photocatalytic & Ag NPs; 99.2% of *E. coli* can be killed within 2 h	170
	AgVO_3_ QDs/g-C_3_N_4_	Sheet-like morphology	*S. aureus, Salmonella*	Photocatalytic generated ROS; killing 96.4% of bacterial within 10 min	171
**BP-based**	BP	215.84 ± 82.09 nm NSs	*E. coli*, *B. subtilis*	ROS-dependent oxidative stress & membrane damage; up to 91.65% and 99.69% for *E. coli* and *B. subtilis*	77
	BP@silk fibroin	About 200 nm layer NSs, silk fibroin modified	*E. coli*, *B. subtilis*	NIR-mediated PTT bactericidal; eliminate most of the bacteria	92
	PPMS/BPS	588 nm NSs, with modified	*E. coli*, *S. aureus*	PDT; 99.3% against *E. coli* and 99.2% against *S. aureus*	122
	AuNPs/BP	Ultrathin NSs	*E. coli*	Catalytic synergistic Au; up to 94.7%	172
**MXenes**	Ti_3_C_2_T_x_	Multilayer transparent flakes, about 400 nm	*E. coli*, *S. aureus*	Physical contact lead to membrane damage; > 98%	173
	MoS_2_/MXene	About 350 nm NSs	*E. coli*, *S. aureus*	Sharp edges lead to membrane damage; >90%	58
	Ti_3_C_2_T_x_ MXene	Single-layer NSs, few hundred nanometers	*E. coli*, *S. aureus*	Inhibit the bacterial growth and efficiently hinder the biofilm formation; > 99% growth inhibition	174
**Others**	B-BiOBr	Square-like NSs	*E. coli*	Photocatalytic generated ROS; Effective	99
	lyso@ZnMgAl-LDH	Flower-like morphology; Lyso modified	*E. coli*, *S. aureus*	LDH enhanced lysozyme antibacterial; fewest bacterial colonies	175
	Ag-LDH	Multilayers platelet-like, 300~500 nm NSs	*E. coli*, *B. subtilis*	Kill the planktonic bacteria and biofilm inhibition; killing almost 100% of bacteria	150
	Sliver/h-BN	Double layer NSs	*Chlorophenols arthrobacter*	Remarkable antibacterial activity	176
	In_2_Se_3_	Multilayer NSs, about 300 nm	*E. coli*	PTT antibacterial; the bacterial inactivation percentage is 98%	177
	RuO_2_	Spherical/sheet like structure, PEG modified	*V. anguillarum, E. tarda, S. iniae & S. parauberis*	Shape dependent direct contact or oxidative stress; Effective	152

**Abbreviations:** BNN6: N,N'-di-sec-butyl-N,N'-dinitroso-1,4-phenylenediamine; PEG: polyethylene glycol; TEG: tetraethylene glycol; *Pseudomonas aeruginosa*: *P. aeruginosa*; PPMS: poly (4-pyridonemethylstyrene); PDDA: polyelectrolyte poly(diallyldimethylammonium chloride); ssDNA: single-stranded DNA; PDMS: polydimethylsiloxane; PDA: polydopamine; LDH: layered double hydroxide.

## Abbreviations

2D NBG: two-dimensional nanomaterials beyond graphene
ATP: adenosine triphosphate
Ar: argon
*B. subtilis*: *Bacillus subtilis*
BP: black phosphorus
B: boron
BNN6: N,N'-di-sec-butyl-N,N'-dinitroso-1,4-phenylenediamine
CFU: colony-forming unit
CB: conduction band
Cys: cysteine
CS: chitosan
CNRGOS_8_: S_8_(core)/rGO(inner shell)/g-C_3_N_4_(outer shell)
DNA: deoxyribonucleic acid
DCFDA: 2',7'-dichlorofluorescin diacetate
*E. coli*: *Escherichia coli*
e^-^: electrons
FLV-MoS_2_: few-layered vertically aligned MoS_2_
Fe_3_O_4_-TNS: Fe_3_O_4_-TiO_2_ nanosheets
GSH: glutathione
GSSG: glutathione disulfide
Gram-: Gram-negative
Gram+: Gram-positive
GNR@LDH: gold nanorod@layered double hydroxide
*g*-C_3_N_4_: graphitic carbon nitride
H_2_O_2_: hydrogen peroxide
h^+^: holes
h-BN: hexagonal boron nitride
ICP-MS: inductively coupled plasma mass spectrometry
LDHs: layered double hydroxides
LAP: laponite
lyso: lysozyme
*MRSA*: *methicillin-resistant S. aureus*
MXenes: transition metal carbides
NIR: near-infrared
NSs: nanosheets
•OH: hydroxyl radicals
^•^O_2_^-^: superoxide anions
^1^O_2_: singlet molecular oxygen
PTT: photothermal therapy
PCT: photocatalytic therapy
PDT: photodynamic therapy
PDDA: poly diallyldimethylammonium
PPMS: poly (4-pyridonemethylstyrene)
*P. aeruginosa*: *Pseudomonas aeruginosa*
PDA: polydopamine
Pen: penicillin
PEG: polyethylene glycol
PPMS: poly (4-pyridonemethylstyrene)
PDDA: polyelectrolyte poly(diallyldimethylammonium chloride)
PDMS: polydimethylsiloxane
PDINH: (perylene-3,4,9,10-tetracarboxylic diimide)
PVDF: polyvinylidene fluoride
QDs: quantum dots
RNA: ribonucleic acid
ROS: reactive oxygen species
rGO-WS_2_: reduced graphene oxide-tungsten disulphide
RGOCNS_8_: S_8_(core)/g-C_3_N_4_(inner shell)/rGO(outer shell)
SEM: scanning electron microscope
ssDNA: single-stranded DNA
*α*-S_8_: cyclooctasulfur
TEM: transmission electron microscope
TEG: tetraethylene glycol
TMDCs: transition metal dichalcogenides
TMDOs: transition metal oxides
TEG: tetraethylene glycol
UV: ultra violet
VB: valence band
